# A Systematic Review and Meta‐Analysis of the Association Between Childhood Maltreatment and Adult Depression

**DOI:** 10.1111/acps.13794

**Published:** 2025-03-03

**Authors:** Christopher B. Watson, Christopher F. Sharpley, Vicki Bitsika, Ian Evans, Kirstan Vessey

**Affiliations:** ^1^ Brain Behaviour Research Group University of New England Armidale New South Wales Australia

**Keywords:** adverse childhood experiences, child abuse, childhood maltreatment, depression, depressive disorders, meta‐analysis, systematic review

## Abstract

**Introduction:**

Childhood maltreatment (CM) and depression are serious global issues with high prevalence and lifelong impacts on physical and mental health. CM has been proposed as a modifiable risk factor for depression that, if prevented, may contribute to a reduction in the global incidence of depressive disorders. Despite this, there is a paucity of reviews examining the strength of the association between these variables. The aim of this systematic review and meta‐analysis was to evaluate the empirical evidence and determine if CM is supported as a preventable risk factor for depression.

**Methods:**

A search was performed in July 2024 for all peer‐reviewed journal articles written in English examining the relationship between CM and adult depression in the electronic databases *EBSCOhost*, *Proquest*, and *Embase*. Studies were included in this review if they measured maltreatment before 18 years of age as the independent variable and adult depression as the dependent variable. Studies were excluded if the outcome variable was grouped with comorbidity and if they did not report primary quantitative data. A total of 77 studies with 516,302 participants met the inclusion criteria for review.

**Results:**

A random‐effects meta‐analysis was used to generate a pooled odds ratio from 87 effect estimates and demonstrated that individuals with a history of any CM are 2.5 times more likely to experience adult depression (*OR* = 2.49 [95% CI: 2.25–2.76]). This increase in odds remained regardless of how the primary studies screened for depression.

**Conclusions:**

These findings confirmed the strong association between the experience of CM and adult depression. High heterogeneity in the meta‐analytic results also suggested that further research is required that applies consistent adjustments for comorbidities and confounding factors and examines the temporal relationship between the variables to establish causality.

**Summary**

- This review demonstrates a strong association between the experience of childhood maltreatment and the development of depression in adulthood.
- This association remains substantial regardless of how depression is measured.
- Findings highlight that childhood maltreatment may play a role as a modifiable risk factor in the complex etiology of depression.
- Most studies included in the review were from high‐income, industrialized countries.
- There was variability in how studies adjusted for confounding and moderating factors.

## 1 Introduction

Depression is a common and distressing health condition that has a complicated etiology and is difficult to measure, diagnose, and treat [1]. Depressive disorders have an estimated 12‐month global prevalence of 5% [2] and reports of lifetime prevalence rates range from 10% in retrospective studies [3] to more than 30% in prospective studies [4]. Depression contributes significantly to the global disease burden and is a leading cause of disability worldwide [5]. It also imposes a substantial economic burden [6] with an approximated worldwide cost nearing US$1 trillion and an estimated loss of 12 billion productive workdays each year [7]. Evidence on the efficacy of treatments for depression is mixed [8] and despite an increase in therapeutic options and availability, there continues to be a treatment‐prevalence paradox where the incidence of depression is increasing [9]. As such, identifying and addressing modifiable developmental risk factors for depression may play an important role in reducing its prevalence [10, 11].

Growing evidence suggests a strong link between exposure to childhood maltreatment (CM) and the development of depression [12, 13]. CM is the experience of any form of abuse (physical, emotional, and sexual), neglect (physical and emotional), or household instability (e.g., parental substance abuse, exposure to intimate partner violence, parental separation, household mental illness, and parental incarceration) before the age of 18 years [14]. Similar to depression, CM is a significant global issue that has lifelong impacts on health and wellbeing [3]. A meta‐analysis of prevalence data from 206 studies across 22 countries estimated that the pooled incidence for a single type of maltreatment was 22.4%, while the experience of four or more types of maltreatment was 16.1% [15]. However, the true global prevalence may be much higher since many countries (particularly low‐ and middle‐income countries) do not have official statistics available [7].

Experiencing CM has been associated with the diagnosis of psychiatric disorders [16], increased health‐risk behaviors [17], and early mortality [18]. In the context of depression, studies have found that individuals with a history of CM endure more severe symptoms, earlier onset, higher recurrence rates, and increased incidence of comorbidities than individuals who have not experienced CM [19, 20]. The relationship between CM and depression is complex, and research suggests it may be influenced by several confounding factors, including genetic and environmental contributors, and moderators such as age, gender, and ethnicity [21, 22, 23, 24]. Nonetheless, the experience of CM appears to be a strong predictor of depression, and it has been proposed that the neurobiological mechanisms underlying this link are alterations to stress response systems [25]. As a form of early‐life stress, CM may contribute to depression through hyperactivation of the hypothalamic–pituitary–adrenal axis response, structural and functional changes in the prefrontal cortex, amygdala, and hippocampus, and an increased immunological inflammatory response [25, 26, 27]. These biological correlates of stress and depression are empirically well supported and provide a strong grounding for an association between CM and adult depression.

CM has been described as an avoidable social problem that could be stemmed by the introduction of preventative policies and practices [3, 28]. Evidence suggests that risk factors for CM are globally consistent but require a dynamic context‐driven response across various social and cultural environments [29, 30]. The WHO [7] advocates for a multisectoral approach to prevention, including caregiver support, education programs, initiatives addressing norms and values, increased child protection laws, and additional support services. This organization also recommends increased international support and global investment [7] which has been recognized in the United Nations Sustainable Development Goals 16.2 [31] and has resulted in several countries implementing national frameworks for CM prevention [32, 33].

**FIGURE 1:**
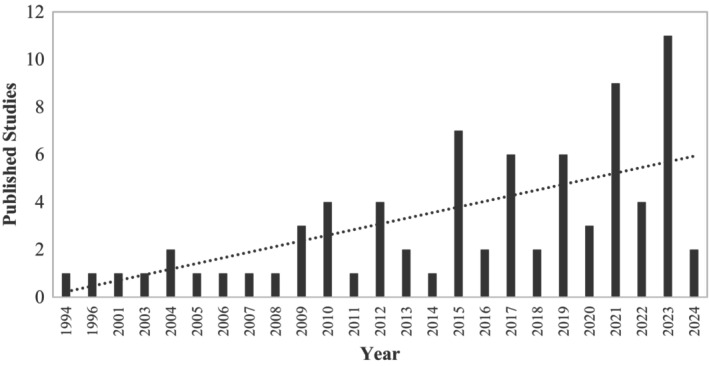
Trend of published studies examining the association between childhood maltreatment and adult depression. This figure shows the growth trend in the number of published studies examining the association between CM and adult depression as identified in the current systematic review.

As an avoidable phenomenon, CM has been proposed as an important modifiable risk factor for adult depression [25]. Yet there is a paucity of reviews and meta‐analyses synthesizing the evidence of this association. An investigation by Nelson et al. [34] found that individuals with any experience of childhood abuse or neglect were almost three times more likely to develop depression in adulthood. However, that review employed a narrow definition of CM that was limited to the two classes of abuse and neglect. Additionally, the primary effect estimates included in that meta‐analysis were not delineated according to the screening method used to measure the variables. As such, the findings of that review may be limited as research suggests there is only a moderate agreement between self‐report measures of depression and diagnostic methods such as structured clinical interview [35, 36, 37]. Studies have shown that estimates of depression prevalence rates are much higher using self‐report scales and that failure to distinguish between screening methods may result in an overestimation of effect [35, 36, 37]. Furthermore, the systematic literature search by Nelson et al. [34] was performed in November 2013, and an updated search in July 2024 indicated that there has been a substantial growth in published studies over this 11‐year period (see Figure 1). As such, an updated synthesis of the evidence for CM as a risk factor for adult depression is necessary.

A systematic review and meta‐analysis by Gardner et al. [38] assessed the effect of five subtypes of CM on the development of anxiety and depression disorders in population‐based samples. That study found a strong relationship between depressive disorders and all CM subtypes that remained regardless of the depression screening method used. Nevertheless, a key limitation of that research was the exclusion of studies that did not report odds ratio or risk ratio effect estimates. This resulted in a low number of estimates being included in some of the meta‐analytic calculations and may also have excluded studies that did not support the association between the variables under investigation. Other reviews have examined the sub‐elements of the association between CM and depression, such as gender differences [23], the timing effect of maltreatment exposure [39], and outcomes experienced by children and adolescents [40], but these analyses do not allow determination of the overall magnitude of the relationship.

The aim of the current review was to address the gaps in the literature by providing an updated systematic and comprehensive examination of the empirical evidence to assess the nature and magnitude of the relationship between CM and adult depression. It was hypothesized that there would be a robust association between the experience of CM and increased odds of developing depression. It was also hypothesized that the estimated effect would be less significant in studies where a diagnostic screening method was used for depression compared with those studies using a self‐report scale or questionnaire. These findings were considered in light of determining if CM prevention might be a theoretically viable approach to reducing the global prevalence of depression.

## 2 Methods

### 2.1 Search Strategy

To identify studies of the association between CM and adult depression, a systematic search was performed in July 2024 for peer‐reviewed journal articles published over the preceding 50 years in the electronic databases *EBSCOhost*, *Proquest*, and *Embase*. The search terms used were “childhood maltreatment,” “child abuse,”, “adverse childhood experiences,” “depression,” “depressive disorder,” “depressive symptoms,” and “major depressive disorder.” Where available, advanced search options were selected to allow each database to apply related words and equivalent terms to the search criteria. In addition, a manual search was undertaken in the reference lists of identified articles, previous reviews, and meta‐analyses. Before the application of inclusion criteria, the first author screened study titles and abstracts for alignment with the research topic.

### 2.2 Selection Criteria

Studies were included in this review if they measured CM before 18 years of age as the independent variable and adult depression (> 18 years) as the dependent variable. No exclusions were made according to the sample used. Studies were excluded if (1) they were not written in English, (2) they were not original, peer‐reviewed journal articles, (3) the outcome variable was grouped with a comorbidity (e.g., depression and anxiety combined), and (4) they did not report primary quantitative data for CM and depression. Reviews, case studies, opinion articles, books, and qualitative studies were not included in this review.

### 2.3 Data Extraction and Quality Assessment

Descriptive information was extracted and entered into an Excel spreadsheet, including the author/s, journal name, year of publication, study location, sample size, number of males and females, source of participants, age (range or mean), measurement of CM, measurement of depression, subtype of CM, and effect size estimates. Where the study measured CM as binary (either present or absent) the subtype was listed as “Any” Whenever possible, adjusted effect estimates were extracted to ensure the impact of CM was measured without confounding variables. The quality of the included studies was evaluated using the *Newcastle‐Ottawa Quality Assessment Scale* (Table S1; [41]). Eleven quality characteristics were assessed, and each was presumed to contribute equally to the overall quality valuation of the study. No minimum quality threshold was set for the inclusion of studies in the review and meta‐analysis; however, ratings were used to perform sensitivity analyses to determine the impact of study quality on the meta‐analysis outcomes.

### 2.4 Statistical Analysis

A meta‐analysis was performed to examine the overall association between the experience of CM and the development of depression in adulthood using effect estimates from the studies that reported CM as a binary variable (present/absent). Additional meta‐analyses were performed according to how the primary studies screened for depression and CM (self‐report scale or a clinical interview) to allow for comparison of the pooled effect sizes. In all instances, odds ratios were selected as the effect estimate for investigation due to the frequency of its use among the reviewed studies (~77%) and to allow for easy interpretation of the probability of depression.

Odds ratios were either obtained directly from the studies or were calculated for studies where sufficient data were available. A random‐effects model was selected for all meta‐analyses to account for between‐study variance. Cochran's Q and *I* ^2^ statistics were estimated to evaluate study heterogeneity [42]. The meta‐analyses were performed using *Stata* statistical software version 15. Multiple sensitivity analyses were undertaken to determine if the overall findings were robust and remained consistent. The undue influence of individual studies on the results of the meta‐analysis was assessed by omitting each study in turn. Furthermore, subgroup analyses were performed by sorting studies according to the screening instrument used for identifying CM and depression. Finally, a meta‐analysis was performed on high‐quality studies only (which were defined as those studies that scored eight points or higher on the *Newcastle‐Ottawa Quality Assessment Scale*). The possibility of publication bias was assessed by viewing funnel plots and performing a trim and fill analysis [43].

## 3 Results

**FIGURE 2:**
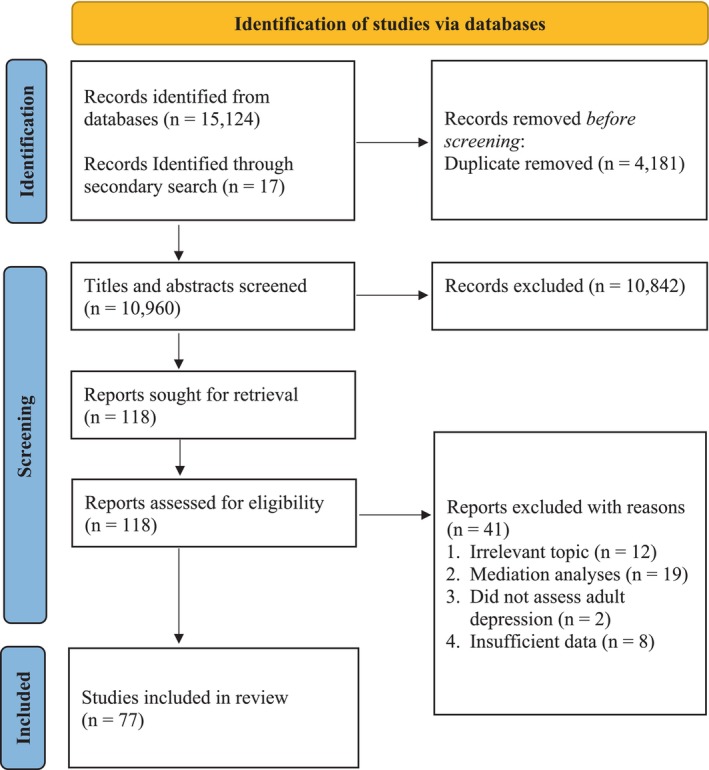
PRISMA flowchart of search and selection process for studies examining the relationship between childhood maltreatment and adult depression.

The process for the identification and selection of studies (*PRISMA* flowchart) is shown in Figure 2. The database search identified 15,124 peer‐reviewed journal articles and a secondary manual search using the reference lists of previous review articles identified an additional 17 studies. The search results included 4181 duplicate reports which were subsequently removed. The titles and abstracts of the remaining 10,960 studies were screened by the first author for alignment with the research topic. After the exclusion of unrelated studies, 118 full‐text articles were retrieved for assessment of inclusion eligibility. A total of 77 studies met the criteria for review and 67 of these reported effect estimates that met the criteria for inclusion in one or more of the meta‐analyses. A detailed description of each of the studies is displayed in Table 1 including all major findings related to adult depression.

**TABLE 1:** Detailed description of studies examining the association between childhood maltreatment and adult depression.

Authors	Year	Journal name	Sample						Test of childhood maltreatment	Test of depression	Type of maltreatment	Depression finding/s
			Location (country)	Total (*N*)	Male	Female	Age (years)	Source				
Afifi et al. [44]	2006	*Child Abuse & Neglect*	USA	5838	2888	2950	15–54	General population	CTS	CIDI	Any physical abuse	Major depression Any: OR = 1.97 (95% CI: 1.59–2.44)* Physical abuse: OR = 1.22 (95% CI: 1.01–1.48)*
Afifi et al. [45]	2009	*Child Abuse & Neglect*	USA	5159	2549	2610	15–54	General population	CTS	CIDI	Any physical abuse Sexual abuse Household instability	Major depression Any: OR = 1.92 (95% CI: 1.29–2.84)* Physical and/or sexual abuse: OR = 2.49 (95% CI: 1.89–3.27) Parental divorce: OR = 1.25 (95% CI: 0.85–1.83)
Al Shawi et al. [46]	2019	*BMC Public Health*	Iraq	401	156	245	18–20	University students	ACE‐Q	DASS	Emotional abuse Emotional neglect Physical abuse Physical neglect Sexual abuse Household instability	Symptoms of depression Emotional abuse: OR = 2.29 (95% CI: 1.01–5.17)* Emotional neglect: OR = 2.78 (95% CI: 1.66–4.64)*** Physical abuse: OR = 1.71 (95% CI: 1.00–2.90)* Physical neglect: OR = 2.13 (95% CI: 1.28–3.53)** Sexual abuse: OR = 2.13 (95% CI: 1.00–4.49)* Exposure to domestic violence: OR = 1.15 (95% CI: 0.67–2.03) Substance abuse household: OR = 2.43 (95% CI: 0.79–7.36) Mental illness in household: OR = 2.64 (95% CI: 1.28–5.42)*
Amone‐P'Olak and Letswai [47]	2020	*South African Journal of Psychiatry*	Botswana	392	179	213	18–32	University students	ACE‐Q	BDI	Any	Symptoms of depression 1–2 ACEs: *M* = 10.68, SD = 9.66 3–4 ACEs: *M* = 13.03, SD = 11.22 ≥ 5 ACEs: *M* = 16.29, SD = 13.69
Angelakis and Gooding [48]	2022	*Psychiatry Research*	United Kingdom	842	151	691	Mean 34.62	General population	Two questions asking to self‐report previous experiences of sexual or physical abuse	CES‐D	Physical abuse Sexual abuse	Symptoms of depression Individuals reporting childhood physical and/or sexual abuse had more severe depression: *x* ^2^ (1, *N* = 842) = 41.47, *p <* 0.001
Babatunde et al. [49]	2024	*Preventative Medicine*	USA	60,122	25,470	34,652	≥ 60	General population	BRFSS‐ACE	Single self‐report question on the history of depression	Any	Diagnosis of a depressive disorder White One ACE: OR = 1.57 (95% CI: 1.26–1.95)* 2–3 ACE: OR = 2.12 (95% CI: 1.72–2.63)* ≥ 4 ACEs: OR = 3.83 (95% CI: 3.07–4.79)* Black One ACE: OR = 1.30 (95% CI: 0.66–2.56) 2–3 ACE: OR = 1.29 (95% CI: 0.66–2.51) ≥ 4 ACEs: OR = 3.39 (95% CI: 1.71–6.71)* Hispanic One ACE: OR = 3.92 (95% CI: 1.55–9.93)* 2–3 ACE: OR = 4.05 (95% CI: 1.54–10.69)* ≥ 4 ACEs: OR = 12.61 (95% CI: 4.75–33.43)*
Berber Celic and Odaci [50]	2019	*International Journal of Social Psychiatry*	Turkey	636	159	477	17–27	University students	CTQ	DASS	Any	Symptoms of depression A positive significant correlation between maltreatment as a child and adult depression (*r* = 0.32, *p* < 0.01)
Bonomi et al. [51]	2008	*Child Abuse & Neglect*	USA	3568	0	3568	18–64	Sample drawn from the membership files of a large health insurance company	BRFSS—ACE	CES‐D	Any	Depressive symptoms: OR = 1.85 (95% CI: 1.53–2.24) Severely depressed: OR = 2.40 (95% CI: 1.84–3.12)
Cannon et al. [52]	2010	*Violence and Victims*	USA	3568	0	3568	18–64	Sample randomly drawn from the membership files of a large health insurance company	BRFSS	CES‐D	Physical abuse Sexual abuse Household instability	Symptoms of depression Childhood abuse: *PR* = 1.55 (95% CI: 1.30–1.85) Childhood abuse and witness IPV: *PR* = 1.96 (95% CI: 1.58–2.43) Symptoms of depression (severe) Childhood abuse: *PR* = 1.63 (95% CI: 1.28–2.08) Childhood abuse and witness IPV: *PR* = 2.55 (95% CI: 1.95–3.34)
Cavanaugh and Nelson [53]	2022	*Journal of Affective Disorders*	USA	6260	2202	4059	20 to 65+	African American adults	ACE‐Q	AUDADIS‐IV	Any	Women past‐year depressive episode Any child abuse/neglect: OR = 2.45 (95% CI: 2.16–2.77) Men past‐year depressive episode Any child abuse/neglect: OR = 2.02 (95% CI: 1.71–2.38)
Chang et al. [17]	2019	*PLoS ONE*	China	1501	453	1048	18–59	General population	ACE‐IQ	CES‐D	Any	Symptoms of depression ACE score: OR = 1.37 (95% CI: 1.27–1.48)
Chapman et al. [54]	2004	*Journal of Affective Disorders*	USA	9460	4352	5108	Mean 56.6	Clinic sample	ACE‐Q	DIS and CES‐D	Any	Lifetime history of depression ACE score 1: OR = 1.3 (95% CI: 1.10–1.50)*** ACE score 2: OR = 1.6 (95% CI: 1.30–2.00)*** ACE score 3: OR = 2.5 (95% CI: 2.00–3.10)*** ACE score 4: OR = 2.4 (95% CI: 1.90–3.20)** ACE score > 5: OR = 3.7 (95% CI: 2.70–5.00)***
Chen et al. [55]	2021	*Frontiers in Psychiatry*	China	30,179	12,688	17,449	Mean 19.9	University students	CTQ‐SF	CES‐D	Physical abuse Emotional abuse Sexual abuse Physical neglect Emotional neglect	Symptoms of depression Physical abuse: OR = 1.20 (95% CI: 1.17–1.23)*** Emotional abuse: OR = 1.21 (95% CI: 1.19–1.23)*** Sexual abuse: OR = 1.19 (95% CI: 1.16–1.22)*** Physical neglect: OR = 1.14 (95% CI: 1.12–1.16)*** Emotional neglect: OR = 1.08 (95% CI: 1.07–1.09)***
Cheong et al. [56]	2017	*BMJ Open*	Ireland	2047	941	985	50–69	Clinic sample	ACE‐Q	CES‐D	Any	Symptoms of depression (later‐life) With ACE: OR = 2.85 (95% CI: 1.64–4.95)***
Comijs et al. [57]	2013	*Journal of Affective Disorders*	Netherlands	508	177	331	60–93	General population, primary care, and mental health care	Childhood abuse inventory	CIDI and IDS	Any	Diagnosis of a depressive disorder Depression in older adults: OR = 7.18 (95% CI: 4.18–12.30)*** Early onset depression: OR = 13.73 (95% CI: 7.31–25.80)*** Middle‐age onset: OR = 5.36 (95% CI: 2.90–9.90)*** Late onset: OR = 4.74 (95% CI: 2.51–8.95)***
Comtois‐Cabana et al. [58]	2023	*PLoS ONE*	Canada	156	156	0	18–35	General population	CTQ‐SF	BDI	Any	Symptoms of depression Child maltreatment was significantly associated with depressive symptoms (*β* = 0.25, *p* < 0.001)
Cong et al. [59]	2012	*Psychological Medicine*	China	4597	0	4597	30–60	Recruited from 53 provincial mental health centers and psychiatric departments of general medical hospitals	Interview with a psychiatrist	CIDI	Sexual abuse	Diagnosis of a depressive disorder Any form of child sexual assault was significantly associated with recurrent major depression: OR = 3.26 (95% CI: 1.95–5.45)***
Cross et al. [60]	2023	*Child Abuse & Neglect*	USA	450	225	225	18–79	Online crowdsourcing network	ACE‐DQ	PHQ‐2	Any	Symptoms of depression Any ACE: OR = 1.48 (95% CI: 1.35–1.62) Diagnosis of a depressive disorder Any ACE: OR = 1.39 (95% CI: 1.28–1.51)
Easton et al. [61]	2019	*Health & Social Work*	USA	479	191	288	Mean 47.4	American Indian Adults	ACE‐Q	PHQ‐9	Sexual abuse	Symptoms of depression Childhood sexual abuse was positively associated with depression in adulthood (*β* = 1.96, *p* < 0.01)
Ege et al. [62]	2015	*The American Journal of Geriatric Psychiatry*	USA	8051	3784	4267	≥ 60	General population	Telephone Interview	PHQ‐8	Physical abuse Sexual abuse Household instability	Symptoms of depression Witnessing IPV: OR = 1.32 (95% CI: 0.65–2.69) Physical abuse: OR = 2.94 (95% CI: 1.68–5.13)* Verbal abuse: OR = 1.11 (95% CI: 0.69–1.76) Sexual abuse: OR = 3.66 (95% CI: 1.01–13.20)*
Gallo et al. [63]	2017	*Journal of Affective Disorders*	Brazil	3715	1761	1954	N/A	Birth cohort	CTQ	MINI‐V	Physical neglect Physical abuse Psychological abuse Sexual abuse Household instability	Diagnosis of a depressive disorder Women Physical neglect: OR = 1.5 (95% CI: 0.80–2.70) Physical abuse: OR = 0.9 (95% CI: 0.50–1.50) Emotional maltreatment; OR = 2.7 (95% CI: 1.90–3.80)** Sexual abuse: OR = 0.7 (95% CI: 0.30–1.70) Exposure to domestic violence: OR = 1.90 (95% CI: 1.20–2.90)** Men Physical neglect: OR = 1.30 (95% CI: 0.50–3.40) Physical abuse: OR = 1.30 (95% CI: 0.50–3.40) Emotional maltreatment: OR = 1.30 (95% CI: 0.60–2.80) Exposure to domestic violence: OR = 1.60 (95% CI: 0.70–3.70)
Ghassemi et al. [64]	2010	*Eastern Mediterranean Health Journal*	Iran	709	0	709	20–45	Mental health seminar attendees	CTS and PAAS	BDI	Any	Diagnosis of a depressive disorder Any abuse: OR = 4.24 (95% CI: 1.94–9.27)
Guerrero et al. [65]	2023	*Multiple Sclerosis Journal—Experimental, Translational and Clinical*	USA	1990	449	1541	18–69	Multiple sclerosis patients	Telephone Interview	Telephone Interview	Any	Symptoms of depression Exposure to any adverse childhood experiences increased the odds of depression in people with multiple sclerosis: OR = 1.71, (95% CI: 1.21–2.42)
Gursoy et al. [66]	2023	*Archives of Psychiatric Nursing*	Turkey	395	50	345	30–61	Nurses	CTQ‐28	DASS	Any	Symptoms of depression Maltreatment correlates with CTQ score (*r* = 0.109, *p* < 0.05)
Hatcher et al. [67]	2019	*Journal of Adolescent Health*	South Africa	2427	2427	0	18–30	Urban community sample	CTQ‐SF	CES‐D	Any	Symptoms of depression Any abuse: OR = 6.78 (95% CI: 5.40–8.17)***
Hayashi et al. [68]	2015	*BMC Psychiatry*	Japan	113	55	58	25–75	Recruited from psychiatric clinics and hospitals	CATS	BDI	Neglect Sexual abuse Punishment Psychological abuse	Symptoms of depression Neglect: *r* = 0.28, *p* < 0.01 Sexual abuse: *r* = 0.15 Punishment: *r* = 0.29, *p* < 0.01 Emotional abuse: *r* = 0.39, *p* < 0.01
Hovens et al. [69]	2012	*Acta Psychiatrica Scandinavica*	Netherlands	1209	408	801	18–65	General population, primary care, and mental health care	NEMESIS	CIDI	Any	Diagnosis of a depressive disorder Any maltreatment: OR = 1.12 (95% CI: 1.03–1.21)***
Kendler et al. [70]	2004	*Psychological Medicine*	USA	1404	0	1404	17–55	Adult twins from state twin registry	Self‐report sexual abuse questionnaire	Interview using the DSM‐III‐R criteria	Sexual abuse	Diagnosis of a depressive disorder Mild to moderate sexual abuse: HR = 1.62 (95% CI: 1.23–2.15) Moderate to severe sexual abuse: HR = 1.64 (95% CI: 1.13–2.39)
Kessler and Magee [71]	1994	*Journal of Health and Social Behaviour*	USA	1024	N/A	N/A	≥ 25	General population	Interview assessing eight indicators of childhood adversity	DIS	Household instability	Major depressive episode Childhood family violence: OR = 1.99 (95% CI: 1.32–3.00)
Khan et al. [72]	2015	*Frontiers in Psychiatry*	USA	560	223	337	18–25	General population	MACE Scale, CTS, CTQ, ACE‐Q	Kellner Symptom Questionnaire, SCL‐90, SIGH‐SAD	Any	Major depressive disorder Any maltreatment: OR = 2.76 (95% CI: 1.73–4.52)
Kim et al. [73]	2013	*BMJ Open*	South Korea	11,526	5143	6383	18 to 60+	General population	Four yes or no questions assessing household instability	CES‐D	Household instability	Symptoms of depression Parental divorce: OR = 2.07 (95% CI: 1.40–3.05)*** Parental death: OR = 1.34 (95% CI: 1.19–1.50)*** Suspension of education due to financial strain: OR = 1.59 (95% CI: 141–1.80)*** Raised by a relative's due to financial strain: OR = 1.64 (95% CI: 1.28–2.04)***
King [74]	2021	*Journal of Affective Disorders*	USA	2430	713	1717	18–87	Online crowdsourcing network	ACE‐Q	Mental Health Diagnostic Panel	Sexual abuse Physical abuse Emotional abuse Household instability	Major depressive disorder Sexual abuse: OR_*M*_ = 3.16, *p* < 0.001 Emotional abuse: OR_*M*_ = 2.62, *p* < 0.001 Physical abuse: OR_*M*_ = 2.41, *p* < 0.001 Maternal battering: OR_*M*_ = 2.15, *p* < 0.001
Kisley et al. [75]	2018	*The British Journal of Psychiatry*	Australia	3778	1988	1790	21	Birth cohort study	Reported maltreatment cases	CES‐D	Any	Symptoms of depression (lifetime) Any maltreatment: OR = 2.33 (95% CI: 1.19–4.58)**
Kisley et al. [76]	2020	*Journal of Psychiatric Research*	Australia	2861	1235	1626	30	Birth cohort study	Reported maltreatment cases	CIDI	Any	Diagnosis of a depressive disorder (lifetime) Any maltreatment: OR = 2.45 (95% CI: 1.20–5.02)**
Kisley et al. [77]	2021	*Journal of Affective Disorders*	Australia	2425	968	1457	30	Birth cohort study	CTQ‐SF Agency notified	CIDI	Any	Diagnosis of a depressive disorder (lifetime) Any self‐reported maltreatment: OR = 2.51 (95% CI: 2.01–3.13)*** Any agency‐reported maltreatment: OR = 1.51 (95% CI: 1.01–2.26)*
Korkeila et al. [78]	2005	*Social Psychiatry and Psychiatric Epidemiology*	Finland	21,101	8543	12,334	20–54	General population	Six‐item self‐report questionnaire about history of maltreatment	BDI	Any	Symptoms of depression Any maltreatment women: OR = 3.09 (95% CI: 2.58–3.70)*** Any maltreatment men: OR = 2.64 (95% CI: 2.13–3.28)***
Korkeila et al. [79]	2010	*Journal of Affective Disorders*	Finland	16,877	6487	10,390	20–54	General population	Six‐item self‐report questionnaire about history of maltreatment	BDI	Any	Symptoms of depression 1 maltreatment: OR = 1.28 (95% CI: 0.98–1.67) 2 maltreatments: OR = 1.99 (95% CI: 1.51–2.64) 3–6 maltreatments: OR = 2.70 (95% CI: 2.10–3.47)
Lara et al. [80]	2015	*Brazilian Journal of Psychiatry (Revista Brasileira de Psiquiatria)*	Mexico	357	0	357	Mean: 27.05	Clinic sample	CECA‐Q	BDI	Sexual abuse Physical abuse Verbal abuse	Symptoms of depression Sexual, physical, and verbal abuse: OR = 3.01 (95% CI: 1.36–6.64)*
Lee and Chen [81]	2017	*Child Abuse & Neglect*	USA	60,598	23,966	36,632	≥ 18	General population	BRFSS‐ACE	PHQ‐8	Any	Diagnosis of a depressive disorder (lifetime) Any maltreatment: OR = 4.91 (95% CI: 3.91–6.16)* Symptoms of depression (current) Any maltreatment: OR = 5.58 (95% CI: 4.32–7.22)*
LeMasters et al. [82]	2021	*BMC Public Health*	Pakistan	889	0	889	Mean 26.7	Birth cohort study	ACE‐IQ	SCID	Any	Major depressive episode Any maltreatment: *PR* = 3.13 (95% CI: 1.73–5.65)
Lereya et al. [83]	2015	*Lancet Psychiatry*	England and USA	5177	2308	2869	N/A	Birth cohort study	Interviews and maternal reports in repeated questionnaires	CIS‐R and YAPA	Any	Diagnosis of a depressive disorder Any maltreatment: OR = 1.40 (95% CI: 0.90–2.20)
Lian et al. [84]	2024	*Journal of Affective Disorders*	Australia	2551	1315	1236	60–66	General population	20‐item questionnaire from PATH project	GDS, PHQ‐9	Any	Symptoms of depression (GDS) Any maltreatment: *β* = 0.15 (95% CI: 0.12–0.17) Symptoms of depression (PHQ‐9) Any maltreatment: *β* = 0.57 (95% CI: 0.47–0.67)
Lin et al. [85]	2023	*Social Psychiatry and Psychiatric Epidemiology*	China	11,408	5466	5582	Mean 59.02	General population	ACE‐Q	CES‐D	Physical abuse Emotional neglect Household instability	Symptoms of depression Threat‐related ACEs: OR = 1.75 (95% CI: 1.49–2.05) Deprivation‐related ACEs: OR = 2.02 (95% CI: 1.67–2.43)
Loxton et al. [86]	2021	*Child Abuse & Neglect*	Australia	8609	0	8609	20–25	Birth cohort study	ACE‐Q	K10	Any	Symptoms of depression Any maltreatment: OR = 2.03 (99% CI: 1.84–2.24)
McFarland et al. [87]	2016	*Psychosomatics*	USA	125	0	125	26–84	Patients with breast cancer	RFQ	HADS	Abuse Neglect Household instability	Symptoms of depression (HADS > 8) Abuse; OR = 0.99 (99% CI: 0.72–1.39) Neglect: OR = 1.34 (99% CI: 1.21–1.58) Chaotic home: OR = 1.01 (99% CI: 0.85–1.21)
Mullen et al. [88]	1996	*Child Abuse & Neglect*	New Zealand	1376	0	1376	18–65	General population	Face‐to‐face interview	PSE‐SF	Any	Symptoms of depression (lifetime) Any maltreatment: OR = 3.67 (95% CI: 2.29–5.87)***
Novelo et al. [89]	2018	*Child Abuse & Neglect*	Brazil	449	161	288	≥ 60	General population	CTQ	Geriatric Depression Scale	Any	Symptoms of depression (any) Any maltreatment: OR = 4.39 (95% CI: 1.30–14.85)** Symptoms of depression (mild to moderate) Any maltreatment: OR = 6.35 (95% CI: 1.75–23.11)** Symptoms of depression (severe) Any maltreatment: OR = 4.08 (95% CI: 0.58–28.77)
Ouellet‐Morin et al. [90]	2015	*Depression and Anxiety*	England and Wales	1052	0	1052	20–48	Twin study	CTQ‐SF	DIS	Any	Diagnosis of a depressive disorder Any maltreatment: OR = 2.64 (95% CI: 1.74–4.01)
Paradis et al. [91]	2009	*Journal of the American Academy of Child and Adolescent Psychiatry*	USA	346	172	174	18–30	General population	Face‐to‐face interview	DIS‐IV	Household instability	Diagnosis of a depressive disorder Family physical violence: OR = 2.00 (95% CI: 0.80–5.30)
Peng et al. [92]	2022	*Journal of Affective Disorders*	China	8014	2853	5161	Mean 58.5	General population	Self‐report questionnaire about the loss of a parent	PHQ‐9	Household instability	Symptoms of depression Childhood parental loss: OR = 1.61 (95% CI: 1.27–2.03)***
Petersen et al. [93]	2022	*Frontiers in Psychiatry*	Germany	2288	1091	1197	25 to 75+	General population	ACE‐Q	PHQ‐4	Any	Symptoms of depression Any maltreatment: OR = 10.20 (95% CI: 8.67–15.55)*
Poole and Dobson [94]	2017	*Child Abuse & Neglect*	Canada	4006	1269	2719	Mean 44.13	Clinic sample	ACE‐Q	PHQ‐9	Any	Symptoms of depression ACE score 1: OR = 1.53 (95% CI: 0.87–2.71) ACE score 2: OR = 3.13 (95% CI: 1.82–5.39) ACE score 3: OR = 4.54 (95% CI: 2.59–7.97) ACE score > 4: OR = 7.25 (95% CI: 4.48–11.72)
Rehan et al. [95]	2017	*PLOS One*	Finland	10,980	3766	7214	Mean 29	General population	CTQ	BSI‐18	Emotional abuse Physical abuse Sexual abuse Emotional neglect Physical neglect	Symptoms of depression Emotional abuse: OR = 3.74 (95% CI: 2.06–6.81)*** Physical abuse: OR = 3.03 (95% CI: 0.99–9.33)* Sexual abuse: OR = 2.40 (95% CI: 1.10–5.21)* Emotional neglect: OR = 4.78 (95% CI: 2.40–9.56)*** Physical neglect: OR = 9.86 (95% CI: 1.99–48.93)***
Reinherz et al. [96]	2003	*The American Journal of Psychiatry*	USA	354	170	184	18–26	General population	Face‐to‐face interview	DIS‐III‐R and DIS‐IV	Any	Diagnosis of a depressive disorder Any maltreatment: OR = 1.60 (95% CI: 0.73–3.53)
Remigio‐Baker et al. [97]	2014	*Maternal and Child Health Journal*	USA	3437	0	3437	18 to 73+	General population	ACE‐Q	PHQ‐8	Any	Symptoms of depression ACE score 1: OR = 2.11 (95% CI: 1.16–3.81) ACE score 2: OR = 2.90 (95% CI: 1.51–5.58) ACE score 3–4: OR = 3.94 (95% CI: 2.13–7.22) ACE score > 5: OR = 4.04 (95% CI: 2.26–7.22)
Roland et al. [98]	2021	*Family Practice*	France	25,319	12,330	12,989	18–75	General population	Health Barometer Telephone Interview	CIDI‐SF	Any	Symptoms of depression (last year) Any maltreatment men: OR = 1.98 (95% CI: 1.57–2.50) Any maltreatment women: OR = 2.25 (95% CI: 1.95–2.60)
Rubino et al. [99]	2009	*The Journal of Nervous and Mental Disease*	Italy	788	339	449	Mean 38.9	Hospital inpatients	The Abuse Questionnaire	SCID‐I	Any	Diagnosis of a depressive disorder Any maltreatment: OR = 3.06 (95% CI: 1.64–5.70)
Rudenstein et al. [100]	2015	*Military Medicine*	USA	991	904	87	25–44	National Guard soldiers	ACE‐Q	PHQ‐9	Any	Symptoms of depression Any maltreatment: OR = 1.90 (95% CI: 1.10–3.10)
Russell et al. [101]	2010	*Child Abuse & Neglect*	USA	1175	658	517	20–24	General population	Self‐report	CES‐D	Household instability	Symptoms of depression Frequent exposure to domestic abuse is a significant risk factor for depressive symptoms in young adulthood (*β* = 1.71, *p* < 0.05)
Saleh et al. [102]	2017	*Psychological Medicine*	USA	129	47	82	20–50	University students	ELSQ	MINI‐5, MADRS	Sexual abuse Physical abuse Emotional abuse Household instability	Diagnosis of a depressive disorder Emotional trauma: *F*_1,121_ = 6.79, *p* = 0.0103 Sexual abuse: *F*_1,121_ = 6.00, *p* = 0.0157 Severe family conflict: *F*_1,121_ = 7.85, *p* = 0.0059
Satinsky et al. [103]	2021	*PLoS Medicine*	Uganda	1626	718	908	17 to 40+	General population	ACE‐IQ	HSCL‐D	Any	Major depressive disorder Any maltreatment: *RR* = 1.19 (95% CI: 1.11–1.27)***
Schilling et al. [104]	2007	*BMC Public Health*	USA	1093	643	682	16 to 20+	General population	Sexual abuse questions based on the ACE‐Q	CES‐D	Sexual abuse	Symptoms of depression Sexual abuse women: *β* = 0.614, *p* < 0.05 Sexual abuse men: *β* = 1.41, *p* < 0.05
Scott et al. [105]	2012	*The British Journal of Psychiatry*	New Zealand	1413	599	814	16–27	General population	NZMHS and NZCYF	CIDI	Any	Diagnosis of a depressive disorder (12‐month) Retrospective reporting; OR = 2.40 (95% CI: 1.43–4.04) Prospective reporting: OR = 2.46 (95% CI: 1.27–4.76) Diagnosis of a depressive disorder (lifetime) Retrospective reporting: OR = 2.51 (95% CI: 1.65–3.83) Prospective reporting: OR = 2.37 (95% CI: 1.42–3.94)
Scott et al. [106]	2023	*Medical Journal of Australia*	Australia	8503	4195	4182	≥ 16	General population	JVQ‐R2	MINI	Any	Major depressive disorder Any maltreatment: OR = 3.19 (95% CI: 2.68–3.80)
Shanahan et al. [107]	2011	*Psychological Medicine*	USA	837	568	436	19–21	General population	CAPA and YAPA	CAPA and YAPA	Sexual abuse Physical abuse	Symptoms of depression (young‐adult onset) Sexual and physical abuse: OR = 2.95 (95% CI: 0.64–13.65)
Taillieu et al. [108]	2016	*Child Abuse & Neglect*	USA	30,307	14,408	15,899	Mean 47.83	General population	ACE‐Q	AUDADIS‐IV	Any	Major depressive disorder Any maltreatment: OR = 1.90 (95% CI: 1.40–2.50)***
Telfar et al. [109]	2023	*Child Abuse & Neglect*	New Zealand	1265	635	630	21–40	Birth cohort study	Face‐to‐face interview	CIDI	Sexual abuse Physical abuse Neglect	Major depressive disorder Sexual abuse: OR = 1.89 (95% CI: 1.31–2.71)*** Physical abuse: OR = 1.80 (95% CI: 0.90–3.59)*** Neglect: OR = 2.30 (95% CI: 1.66–3.18)***
Tracy et al. [110]	2019	*Depression and Anxiety*	United Kingdom	9665	4983	4682	18	General population	ACE‐based self‐report questions	CIS‐R, SMFQ	Any	Symptoms of depression (at 18 years) Stable mild maltreatment: OR = 1.22 (95% CI: 0.94–1.58) Decreasing from moderate to mil maltreatment: OR = 1.72 (95% CI: 1.19–2.48) Increasing from mild to high maltreatment: OR = 1.81 (95% CI: 1.15–2.86) Stable high maltreatment: OR = 1.80 (95% CI: 1.00–3.23)
Van Overloop et al. [111]	2023	*Community Mental Health Journal*	USA	20,345	8251	12,134	Mean 56.4	General population	BRFSS‐ACE	PHQ‐8	Any	Symptoms of depression Any maltreatment: OR = 8.8 (95% CI: 5.6–13.8)
Waite and Shewokis [112]	2012	*Association of Black Nursing Faculty Journal*	USA	796	154	647	18–88	Clinic sample	ACE‐Q	Health Appraisal Questionnaire—Self‐report	Emotional abuse Physical abuse Sexual abuse Emotional neglect Physical neglect Household instability	Symptoms of depression Emotional abuse: OR = 2.99 (95% CI: 2.03–4.58)*** Physical abuse: OR = 2.96 (95% CI: 2.19–3.98)*** Sexual abuse: OR = 2.82 (95% CI: 2.07–3.18)*** Emotional neglect: OR = 2.57 (95% CI: 1.92–3.44)*** Physical neglect: OR = 1.24 (95% CI: 0.93–1.66) Battered mother: OR = 1.40 (95% CI: 1.06–1.86)* Household substance abuse: OR = 1.89 (95% CI: 1.37–2.62)*** Mental illness in household: OR = 2.53 (95% CI: 1.89–3.36)*** Parental separation/divorce: OR = 3.30 (95% CI: 2.44–4.46)*** Criminal household member: OR = 1.27 (95% CI: 0.95–1.71)
Wajid et al. [113]	2020	*Archives of Women's Mental Health*	Canada	636	0	636	18–45	Pregnant women recruited from antenatal clinics	ACE‐Q	MINI, EPDS	Any	Prenatal depression ACE score of 4 or higher: OR = 2.41 (95% CI: 1.31–4.44)***
Whitaker et al. [114]	2021	*BMC Public Health*	USA	4344	2009	2335	Mean 54.1	General population	ACE‐Q	CIDI‐SF	Any	Major depressive episode ACE score 1: OR = 1.16 (95% CI: 0.86–1.57) ACE score 2: OR = 2.01 (95% CI: 1.49–2.72) ACE score 3–5: OR = 2.34 (95% CI: 1.67–3.28)
Wise et al. [115]	2001	*The Lancet*	USA	732	0	732	36–45	General population	Interview Survey of Interpersonal Relationships	SCID	Any	Major depressive disorder Any maltreatment: OR = 3.40 (95% CI: 2.40–5.10)
Xiang and Wang [116]	2021	*International Journal of Geriatric Psychiatry*	USA	16,946	7524	9422	Mean 65.4	General population over 51 years	PLQ	CIDI‐SF	Physical abuse Household instability	Major depressive disorder Physical abuse: *SHR* = 1.67 (95% CI: 1.49–1.89)*** Repeated a school year: *SHR* = 1.03 (95% CI: 0.92–1.15) Parental alcohol or drug abuse: *SHR* = 1.11 (95% CI: 1.01–1.23) In trouble with the police: *SHR* = 1.31 (95% CI: 1.13–1.54)** Financial difficulties: *SHR* = 1.08 (95% CI: 0.97–1.21) Received relative help (financial difficulties): *SHR* = 1.17 (95% CI: 1.05–1.31)
Ye et al. [117]	2023	*Journal of Affective Disorders*	China	29,311	13,410	15,901	Mean 20.5	University students	Three self‐report questions on sexual abuse history	CES‐D	Sexual abuse	Symptoms of depression Any sexual abuse: *PR* = 1.26 (95% CI: 1.21–1.31)
Yin et al. [118]	2023	*Frontiers in Public Health*	China	4823	1705	3118	Mean 63.81	Individuals with cardiovascular disease	ACE‐IQ	CES‐D	Any	Symptoms of depression Any maltreatment: OR = 1.23 (95% CI: 1.09–1.38)***
Zhang et al. [119]	2023	*Journal of Affective Disorders*	China	14,484	6925	7559	Mean 60.66	General population	ACE‐IQ	CES‐D	Any	Symptoms of depression ACE score 1: OR = 1.24 (95% CI: 1.07–1.43) ACE score 2: OR = 1.54 (95% CI: 1.32–1.78) ACE score 3: OR = 2.03 (95% CI: 1.72–2.40) ACE score > 4: OR = 2.65 (95% CI: 2.21–3.16)

*Note:* *p < 0.05, **p < 0.01, ***p < 0.001. ACE‐Q: Adverse Childhood Experiences Questionnaire [14]; ACE‐DQ: Adverse Childhood Experiences Dimensions Questionnaire [60]; ACE‐IQ: Adverse Childhood Experiences International Questionnaire [120]; AUDADIS‐IV: Alcohol Use Disorder and Associated Disabilities Interview Schedule‐IV [121]; BDI: Beck Depression Inventory [122]; BRFSS‐ACE: Behavioral Risk Factor Surveillance System ACE Module; BSI‐18: Brief Symptom Inventory 18 Item [123]; CAPA: Child and Adolescent Psychiatric Assessment [124]; CATS: Child Abuse and Trauma Scale [125]; CCMS: Comprehensive Child Maltreatment Scale [126]; CECA‐Q: Childhood Experience of Care and Abuse Questionnaire [127]; CES‐D: Centre for Epidemiological Studies Depression [128]; CIDI: Composite International Diagnostic Interview [129]; CIDI‐SF: Composite International Diagnostic Interview Short Form [130]; CIS‐R: The Clinical Interview Schedule Revised [131]; CTQ: Childhood Trauma Questionnaire [132]; CTQ‐SF: Childhood Trauma Questionnaire Short Form [133]; CTS: Conflict Tactic Scales [134]; DASS: Depression, Anxiety, and Stress Scales [135]; DID: Diagnostic Inventory for Depression [136]; DIS: Diagnostic Interview Schedule; ELSQ: Early Life Stress Questionnaire; EPDS: Edinburgh Postnatal Depression Scale [137]; GDS: Goldberg Depression Scale [138]; HADS: Hospital Anxiety and Depression Scale [139]; HSCL‐D: Hopkins Symptom Checklist for Depression [140]; IDS: Inventory of Depressive Symptoms [141]; JVQ‐R2: Juvenile Victimization Questionnaire Revision 2 [142]; K10: Kessler Psychological Distress Scale [143]; MACE Scale: Maltreatment and Abuse Chronology of Exposure Scale [144]; MADRS: Montgomery–Asberg Depression Rating Scale [145]; MINI: Mini International Neuropsychiatric Interview; NEMESIS: Netherlands Mental Health Survey and Incidence Study; NZCYF: New Zealand Child; NZMHS: New Zealand Mental Health Survey Youth and Family Agency; PAAS: Pregnancy Abuse Assessment Screen [146]; PHQ‐4: Patient Health Questionnaire 4 Item [147]; PHQ‐8: Patient Health Questionnaire 8 Item [148]; PHQ‐9: Patient Health Questionnaire 9 Item [149]; PLQ: Psychosocial and Lifestyle Questionnaire [150]; PSE‐SF: Present State Examination Short Form [151]; RFQ: Risky Families Questionnaire [152]; SCID: Structured Clinical Interview for the Diagnostic and Statistical Manual of Mental Disorders; SCL‐90: Symptom Checklist 90; SDS: Zung Self‐rating Depression Scale [153]; SMFQ: Short Moods and Feelings Questionnaire [154]; SIGH‐SAD: The Structured Interview Guide to the Hamilton Depression Scale with Seasonal Addendum [155]; YAPA: Young Adult Psychiatric Assessment [156].

There were a total of 516,302 participants across the 77 reviewed studies, with the sample sizes ranging from 113 individuals in the smallest study to 60,598 in the largest. The median sample size for all studies was 2288 participants. Just over 40% of the studies (31/77) were conducted in the United States of America, with the remainder occurring in Australia, Botswana, Brazil, Canada, China, Finland, France, Germany, Iran, Iraq, Ireland, Italy, Japan, Mexico, Netherlands, New Zealand, Pakistan, South Africa, South Korea, Turkey, Uganda, and the United Kingdom. Approximately 69% (53/77) of the studies were published in the last 10 years. The earliest report was published in 1994 and the most recent in 2024. The average score on the *Newcastle‐Ottawa Quality Assessment Scale* was 6.79, with results ranging from 4.00 to 9.00 (see Table S1).

There was variability in the moderating and confounding factors assessed by the reviewed studies. Models that adjusted for the sex and age of the participants were the most common (71.4%), followed by participants' education level (50.6%), income (29.8%), ethnicity (28.5%) and marital status (27.3%). A limited number of studies assessed the influence of empirically supported confounders of CM and depression, including income/socioeconomic status (41.5%), chronic illness (6%), body mass index (6%), and negative affectivity/neuroticism (5%). None of the 77 studies examined genetic predisposition as a confounding factor.

### 3.1 CM Measurement

The majority of the investigations included in this review used self‐report screening methods to measure CM (66 out of 77 studies). The most utilized test was the *Adverse Childhood Experiences Questionnaire* (ACE; [14]) which was administered in ~39% of reviewed studies (30/77) either in its original form or as an international variant. The ACE questionnaire is a brief and well‐validated instrument that is widely used in research and clinical practice as a screening tool for CM and as a risk index for future negative health outcomes [157]. The scale is based on the cumulative risk model of CM, which proposes that the lifetime risk of poor health outcomes is positively associated with an accumulation of adverse childhood experiences [158]. The ACE is a yes/no 10‐item self‐report questionnaire evaluating an individual's recall of childhood exposure to abuse, neglect, and household instability [14, 157]. The number of yes responses is totaled to provide the CM accumulation index, which is subsequently interpreted as the risk score.

Another popular self‐reporting screening tool was the Childhood Trauma Questionnaire (CTQ) which was used in 11 of the 77 reviewed studies. Like the ACE scale, the CTQ has been assessed as highly reliable and well‐validated and has questions that delineate between each of the five subtypes of CM [157]. However, it differs from the ACE by using a five‐point Likert scale rather than dichotomous (yes/no) items and by including a validity subscale of three items that assess the reliability of the information reported by the participants [157]. Other self‐reporting screening methods used included the Maltreatment and Abuse Chronology of Exposure Scale (MACE), the Child Abuse and Trauma Scale (CATS), and the Conflict Tactic Scales (CTS). Aside from these, two of the reviewed studies used primary care records from child protection agencies, and nine studies used clinical interviews administered by trained psychologists or psychiatrists.

### 3.2 Depression Measurement

Fewer than half of the reviewed studies screened for depression using clinical interviews (28/77) administered by trained professionals based on the diagnostic criteria for depressive disorders set by the *Diagnostic and Statistical Manual of Mental Disorders* (5th ed., text rev.; DSM‐5‐TR; American Psychiatric Association [APA], [121]). The remaining studies (49/77) used self‐report scales or questionnaires to assess depressive symptoms. The short form (10 items) of the *Centre for Epidemiological Studies Depression* scale (CES‐D; [128]) was the most frequently used self‐report measure, which was utilized in 15 of the 77 analyses. The CES‐D has been assessed as having high validity and internal consistency in both its long and short forms [159, 160]. Other frequently used screening tools were the *Patient Health Questionnaire* (10/77; [149]) and the *Beck Depression Inventory* (7/77; [122]). While the diagnostic interview is recommended as the ‘gold standard’ for the assessment of depression by the DSM‐5‐TR [121], evidence suggests that the above retrospective scales have a strong correlation to the DSM‐5‐TR criteria and comparable reliability and internal consistency [161, 162]. Nonetheless, research suggests that the use of self‐report screening methods for depression can produce poor consistency with diagnostic measures and often overestimates the prevalence of depressive symptoms [35, 36, 37].

### 3.3 The Association Between CM and Adult Depression

**FIGURE 3:**
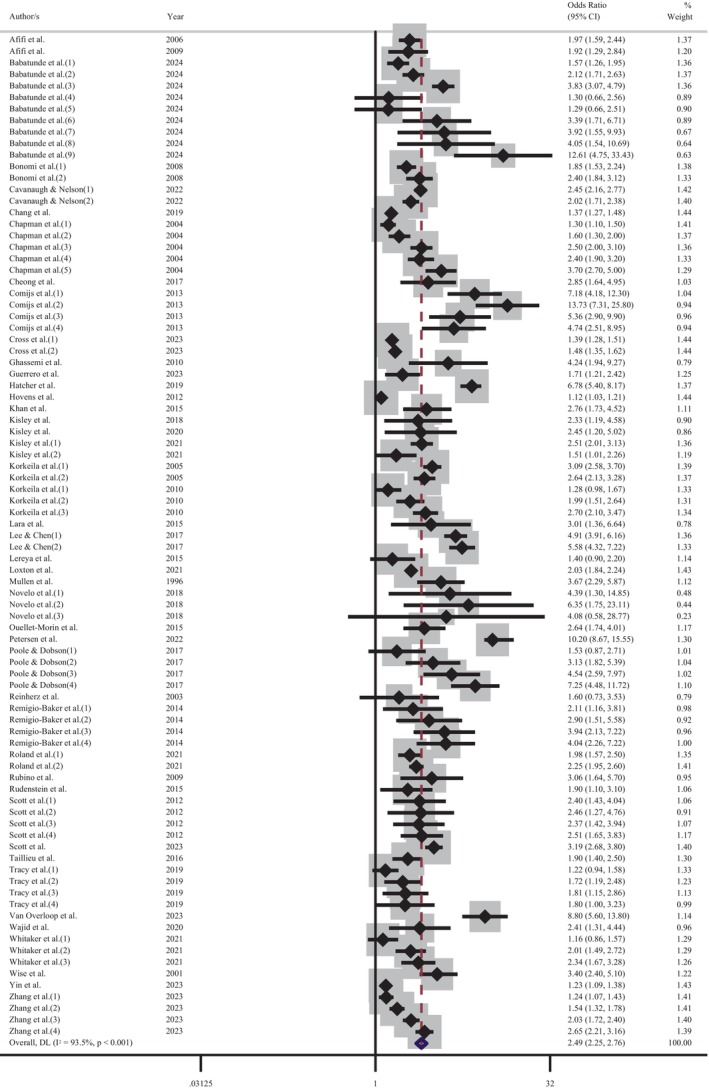
Meta‐analysis of the association between the experience of childhood maltreatment and adult depression.

The 77 studies included in this review reported 101 effect estimates of the relationship between CM as an independent variable and adult depression as a dependent variable. Each of these demonstrated that exposure to CM increased the likelihood of experiencing adult depression. The lowest increase in odds was 1.1 times [69] while the largest was 12.6 times the probability [49]. The random‐effects meta‐analysis of the association between any CM and adult depression included 87 effect estimates, which were drawn from 44 studies (see Figure 3). The results of this analysis indicated that individuals with a history of any CM are approximately two‐and‐a‐half times more likely to experience adult depression (OR = 2.49 [95% CI: 2.25–2.76]) than individuals without a history of CM.

**FIGURE 4:**
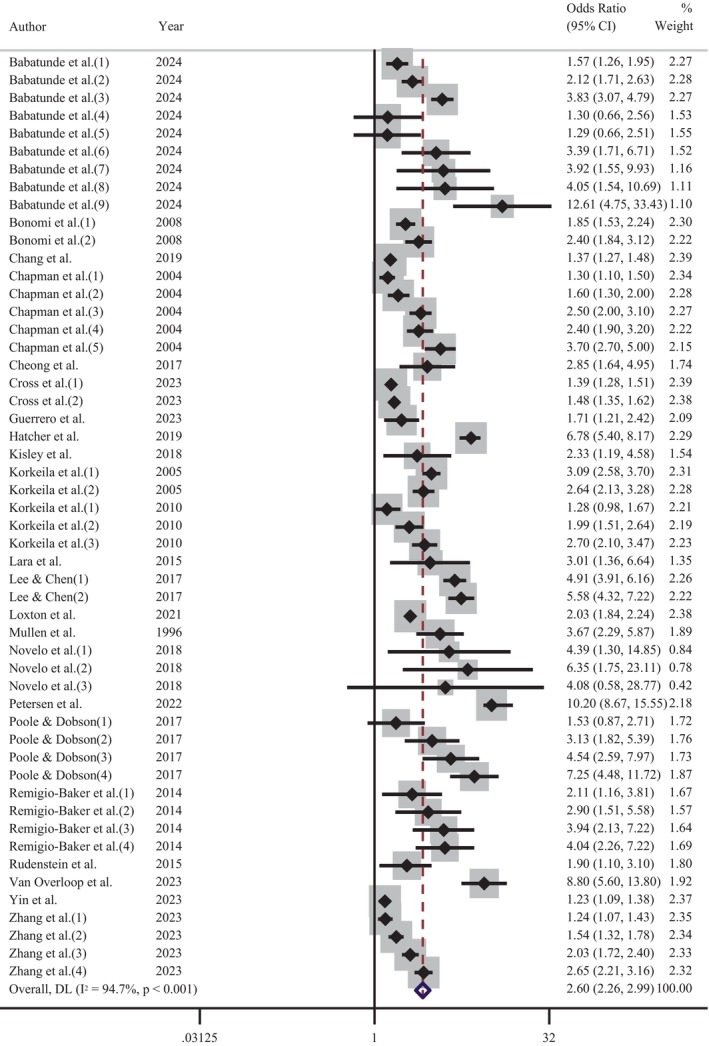
Meta‐analysis of the association between the experience of childhood maltreatment and adult depression (self‐reported).

**FIGURE 5:**
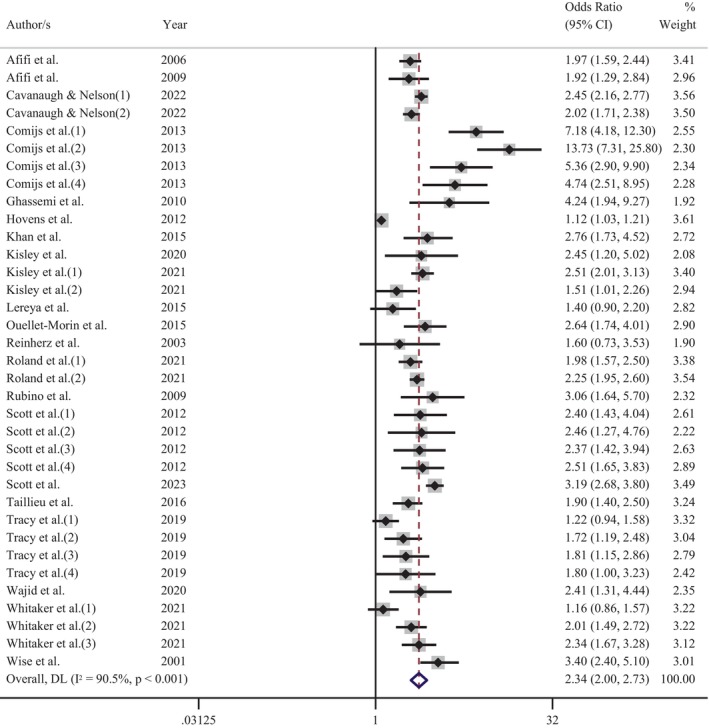
Meta‐analysis of the association between the experience of childhood maltreatment and adult depression (diagnosed in clinical interview).

**FIGURE 6:**
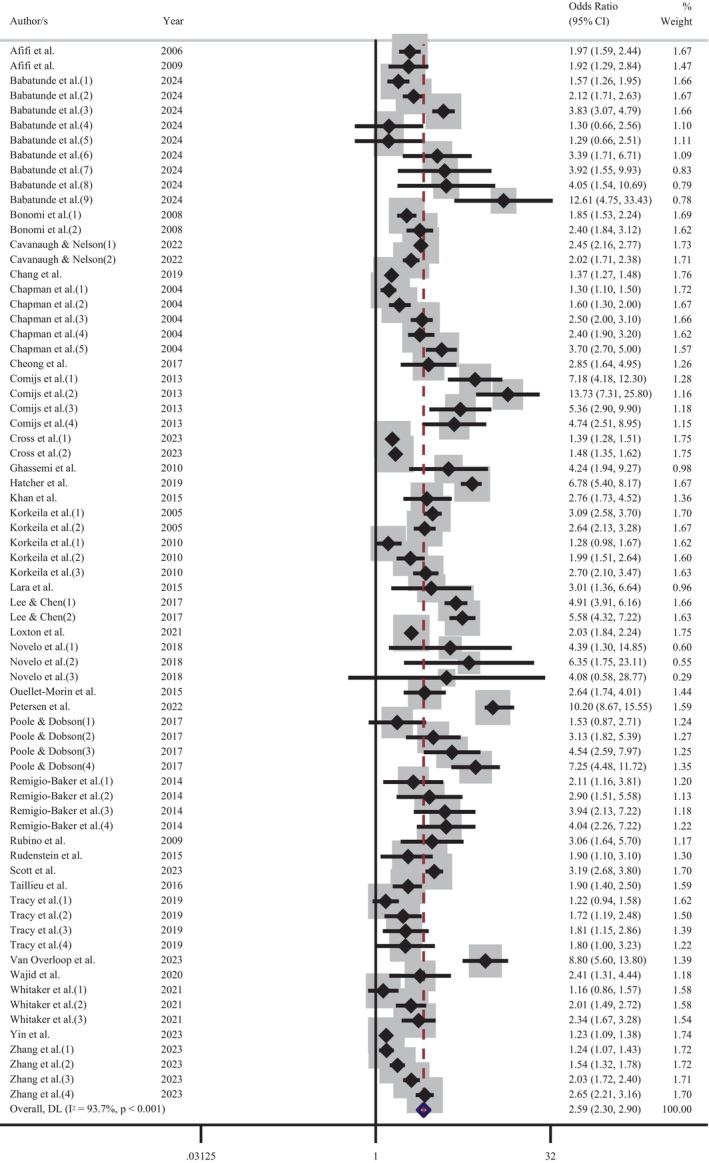
Meta‐analysis of the association between childhood maltreatment (self‐reported) and adult depression.

**FIGURE 7:**
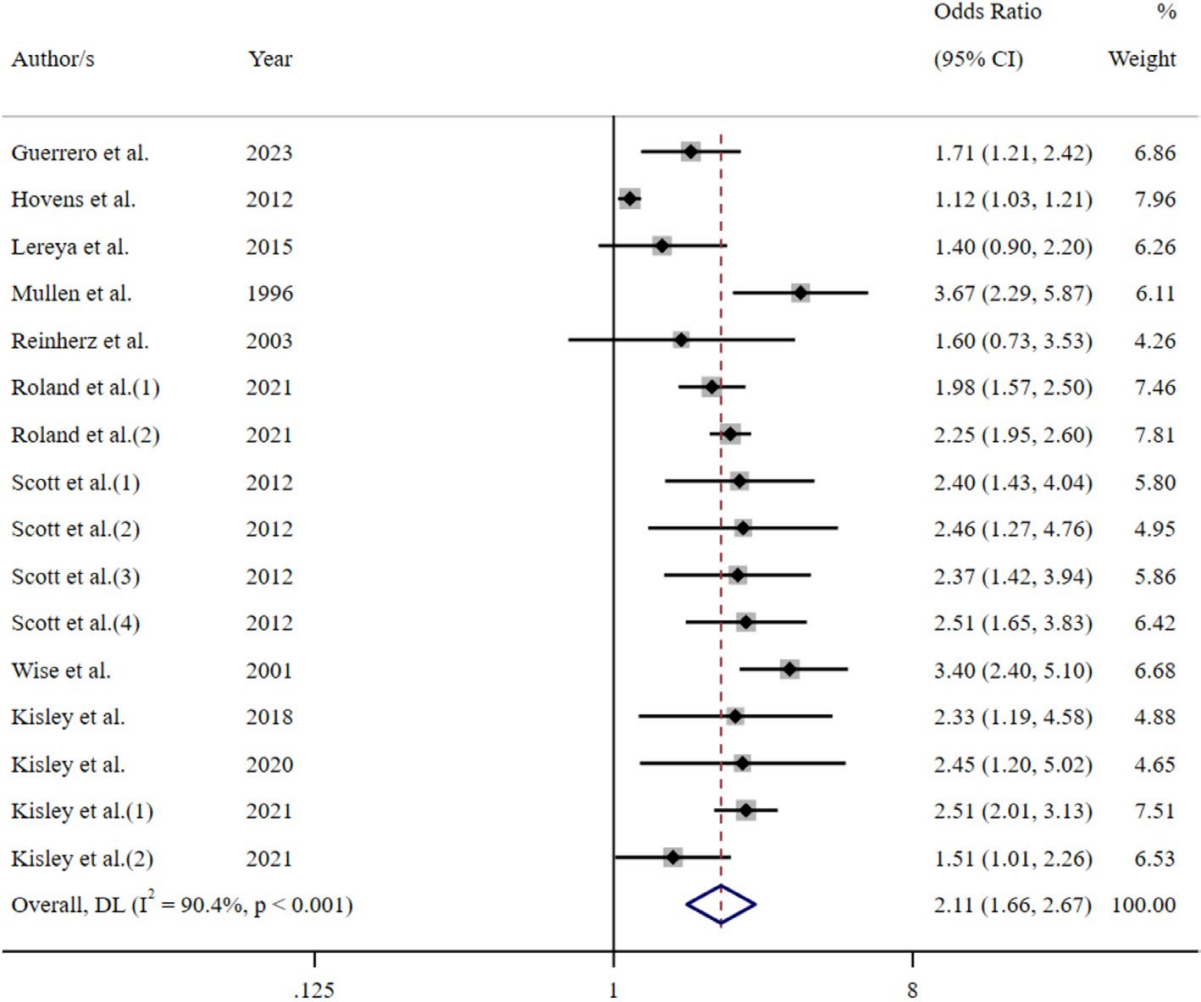
Meta‐analysis of the association between childhood maltreatment (clinical interview) and adult depression.

Further meta‐analyses were performed to determine the influence of depression and CM screening methods on the pooled effect size. An analysis of those studies that screened for depression using self‐report measures indicated that the experience of CM increased the odds of adult depression by 2.6 times (OR = 2.60 [95% CI: 2.26–2.99]; Figure 4). Comparatively, a meta‐analysis of reports that used a diagnostic clinical interview demonstrated an increase of odds 2.3 times (OR = 2.34 [95% CI: 2.00–2.73]; Figure 5). The proximity of these odds ratios for self‐report versus clinical interview suggests that, while self‐report measures of depression may result in a slight overestimation of the effect, the substantial association between the CM and adult depression remains evident. Similarly, exploration of CM screening methods demonstrated that the pooled effect size of studies using a self‐report questionnaire (OR = 2.58 [95% CI: 2.30–2.90]; Figure 6) was marginally higher, but not significantly different than those studies using a clinical interview (OR = 2.13 [95% CI: 1.64–2.77]; Figure 7).

There was significant heterogeneity in the overall meta‐analysis (*I* ^2^ = 93.5%; Cochran's *Q* = 1319.02, df = 86, *p* < 0.001) which suggests that the reported samples differed substantially between studies. This heterogeneity remained after the studies were grouped according to CM screening method (self‐report: *I* ^2^ = 93.7%; Cochran's *Q* = 1118.95, df = 70, *p* < 0.001, clinical interview: *I* ^2^ = 90.4%; Cochran's *Q* = 156.15, df = 15, *p* < 0.001) and depression measure (self‐report: *I* ^2^ = 94.7%; Cochran's *Q* = 961.24, df = 51, *p* < 0.001, diagnostic interview: *I* ^2^ = 90.5%; Cochran's *Q* = 357.69, df = 34, *p* < 0.001). These outcomes confirmed the choice to use a random‐effects model for the meta‐analysis to account for between‐study variance. Further examination of the data was undertaken to evaluate the influence of each independent study on the collective findings. Eighty‐seven permutations of the meta‐analysis were computed, omitting one study at a time, using the *Stata* statistical software (see Figure S1). These calculations demonstrated that no individual study had an undue influence on the pooled effect estimate of the meta‐analysis.

The subgroup analysis of CM screening instruments showed minimal variance between the pooled effect estimates (see Table S2). The ACE questionnaire was the most popular CM self‐report tool, and it demonstrated only a slightly higher pooled effect estimate (OR = 2.36 [95% CI: 2.08–2.66]) than clinically administered interviews (OR = 2.18 [95% CI: 1.77–2.70]). The analysis of depression screening subgroups indicated that the pooled effect estimate from studies that used diagnostic interviews was slightly higher (OR = 2.27 [95% CI: 1.95–2.66]) than the most popular self‐report instrument, the CES‐D (OR = 2.11 [95% CI: 1.72–2.59]; Table S3). The sensitivity analysis of studies identified as high‐quality revealed that CM increased the odds of adult depression by 2.36 times (OR = 2.36 [95% CI: 2.06–2.71]; Figure S2). This pooled effect estimate is slightly lower than the primary meta‐analysis (OR = 2.49 [95% CI: 2.25–2.76]).

## 4 Discussion

This study addressed the limitations of previous research by providing a comprehensive review and meta‐analysis of the association between CM and adult depression. The findings confirmed that experiencing CM increased the odds of developing depression in adulthood by just under 2.5 times. These results are consistent with previous systematic reviews and meta‐analyses [23, 34, 38, 39, 40]; however, the current study further established that the magnitude of this association substantially remained regardless of whether depression was self‐reported or diagnosed by clinical interview. Research has suggested that reliance on self‐report measures for depression can overestimate prevalence and subsequently positively skew meta‐analytic results [35, 36]. However, in the current study, the subgroup analysis stratified by depression screening method indicated that studies that employed a diagnostic interview estimated slightly higher odds than the most popular self‐report questionnaire. Comparatively, the CM screening method used slightly influenced the strength of the pooled effect estimate. Studies that identified CM using a clinical interview demonstrated a lower pooled effect estimate (OR = 2.13 [95% CI: 1.64–2.77]) than studies that used a self‐report method (OR = 2.58 [95% CI: 2.30–2.90]). This outcome is contrary to previous research which found a strong synergy between interviewer‐rated retrospective reports and self‐report questionnaires [163]. As such, it may be an important focus of future research to establish the inter‐rater reliability of CM screening methods.

Other meta‐analyses have determined marginally higher pooled effect sizes than the current study. Nelson et al. [34] found that CM nearly tripled the risk of adult depression (OR = 2.81 [95% CI: 2.35–3.36]) in their assessment of studies published before 2013. However, that study restricted the definition of CM to abuse or neglect and did not include exposure to household instability, which limits the direct comparison of outcomes from that study with those found here. Similarly, Li et al. [39] identified a statistically significant relationship between CM and depression (*r* = 0.17 [95% CI: 0.15–0.18], *p* < 0.0001); however, less than half of the studies included in that analysis were of adult populations. In their meta‐analysis of studies published before March 2018, Gardner et al. [38] found results consistent with the current study (OR = 2.48 [95% CI: 2.14–2.87]). However, that investigation included predominantly children and adolescent samples and had a low number of primary effect estimates (22) included in that meta‐analysis in comparison to the current study. Subsequently, the findings of the current analysis provide a unique and updated evaluation of the empirical evidence supporting the association between CM and adult depression.

Despite the comprehensive nature of this review, the results should be considered in the context of potential limitations. First, there was variability across the reviewed studies in the analysis of moderating and confounding variables. While the majority of studies adjusted for sociodemographic factors, empirically supported confounders, such as socioeconomic status, genetic predisposition, and parental psychopathology, were not consistently considered. Subsequently, the contribution of CM to the development of adult depression may be overestimated given depression's complex etiology. Second, the findings may have limited generalizability due to the elevated number of samples from high‐income, industrialized countries. For example, investigations from the United States of America represented 40% of the reviewed studies and 39% of the effect estimates included in the meta‐analyses. There were also a number of studies that used convenience sampling, such as university students, clinical participants, and employees, which may attract volunteer bias and limit the significance of findings to the broader population. Finally, there was inconsistency in study design and variable measurement across studies that precluded some effect estimates from being included in the overall analysis. The majority of reports used a retrospective design (66 out of 77 studies), while only two studies used primary care records from a child protection agency to confirm participant experience of CM. Nonetheless, nine of the reviewed studies used a prospective design and reported findings that were similar to the overall meta‐analysis outcomes or demonstrated a stronger association.

There were also limitations that should be considered when evaluating the results of the systematic review and meta‐analyses. The selective publication of studies with significant findings and the underreporting of null findings may introduce bias and influence the outcomes of the analysis. Furthermore, the exclusion of non‐English language studies may also introduce bias. There was high heterogeneity in the meta‐analyses, which indicated variability between the included studies. This is consistent with previous reviews of the association between CM and depression [22, 34] and may be a consequence of the complex interplay of contributors involved in the development of depression. While several sensitivity analyses were undertaken in an attempt to explain this heterogeneity, it is proposed that the inconsistencies in how effect estimates were adjusted for moderators may play an important role. It should also be noted that while the findings of this review demonstrate a strong and consistent association between CM and adult depression, the majority of included studies were cross‐sectional and cannot support the conclusion of a causal relationship. Causality requires a clear temporal association and analytic adjustment for numerous covariates. Instead, it is proposed that the findings indicate that CM may be one of many important modifiable contributors to the development of depression.

The findings of this review have important implications for future research and may contribute to the understanding of the complex etiology of depression. In a clinical context, the findings suggest that early intervention and preventative strategies for depression may benefit from the inclusion of treatment strategies and support processes for survivors of CM. Further research is required examining the contribution of CM to depression in the context of the many others empirically supported confounding and moderating factors. Evidence is also required from low‐income, developing countries to complement the well‐established findings of high‐income, industrialized countries. There is an opportunity for longitudinal research examining the temporal association between CM and depression to aid in the determination of a causal relationship. There is also an opportunity to examine the relationship between the different forms of maltreatment and the symptom‐based subtypes of depression that may allow targeted treatment models. Finally, future studies should aim for consistency in the measurement of CM and depression, and a comprehensive approach to adjusting for moderating and confounding variables.

## 5 Conclusions

Depression is a highly prevalent and debilitating health condition that continues to increase in incidence despite improved treatment approaches and broader therapeutic access. There is strong evidence suggesting that the experience of CM may be one of the many factors contributing to the development of depression in adulthood. As a preventable phenomenon, CM may play an important role among these contributors as a modifiable risk factor for depression. The findings of this systematic review and meta‐analysis suggest that CM more than doubles the odds of adulthood depression. These results extend the findings of previous research by establishing that this association persists regardless of the screening methods used.

## Author Contributions

C.B.W. conceptualized the systematic review design, developed the search strategy, extracted data, and carried out the statistical analyses. C.B.W. and C.F.S. collaborated in the article writing. V.B., I.E., and K.V. contributed to developing and finalizing the manuscript.

## Conflicts of Interest

The authors declare no conflicts of interest.

### Peer Review

The peer review history for this article is available at https://www.webofscience.com/api/gateway/wos/peer‐review/10.1111/acps.13794.

## Supporting information


**Data S1.** Supporting Information.

## Data Availability

The data that support the findings of this study are available from the corresponding author upon reasonable request.

## References

[1] H. Herrman , V. Patel , C. Kieling , et al., “Time for United Action on Depression: A Lancet‐World Psychiatric Association Commission,” Lancet 399, no. 10328 (2022): 957–1022, 10.1016/S0140-6736(21)02141-3. 35180424

[2] D. Moreno‐Agostino , Y.‐T. Wu , C. Daskalopoulou , M. T. Hasan , M. Huisman , and M. Prina , “Global Trends in the Prevalence and Incidence of Depression: A Systematic Review and Meta‐Analysis,” Journal of Affective Disorders 281 (2021): 235–243, 10.1016/j.jad.2020.12.035. 33338841

[3] World Health Organization , Responding to Child Maltreatment: A Clinical Handbook for Health Professionals (World Health Organization, 2022).

[4] W. Marx , B. W. J. H. Penninx , M. Solmi , et al., “Major Depressive Disorder,” Nature Reviews. Disease Primers 9, no. 1 (2023): 44, 10.1038/s41572-023-00454-1. 37620370

[5] E. M. Abrams , B. Akombi , S. Alam , et al., “Global Burden of 369 Diseases and Injuries in 204 Countries and Territories, 1990–2019: A Systematic Analysis for the Global Burden of Disease Study 2019,” Lancet 396, no. 10258 (2020): 1204–1222, 10.1016/S0140-6736(20)30925-9. 33069326 PMC7567026

[6] L. Gutierrez‐Rojas , A. Porras‐Segovia , H. Dunne , N. Andrade‐González , and J. A. Cervilla , “Prevalence and Correlates of Major Depressive Disorder: A Systematic Review,” Revista Brasileira de Psiquiatria 42, no. 6 (2020): 657–672, 10.1590/1516-4446-2020-0650. 32756809 PMC7678895

[7] World Health Organization , World Mental Health Report: Transforming Mental Health for All (World Health Organization, 2022).

[8] P. Cuijpers , H. Noma , E. Karyotaki , C. H. Vinkers , A. Cipriani , and T. A. Furukawa , “A Network Meta‐Analysis of the Effects of Psychotherapies, Pharmacotherapies and Their Combination in the Treatment of Adult Depression,” World Psychiatry 19, no. 1 (2020): 92–107, 10.1002/wps.20701. 31922679 PMC6953550

[9] J. Ormel , S. D. Hollon , R. C. Kessler , P. Cuijpers , and S. M. Monroe , “More Treatment but no Less Depression: The Treatment‐Prevalence Paradox,” Clinical Psychology Review 91 (2022): 102111, 10.1016/j.cpr.2021.102111. 34959153

[10] K. E. Cairns , M. B. H. Yap , P. D. Pilkington , and A. F. Jorm , “Risk and Protective Factors for Depression That Adolescents Can Modify: A Systematic Review and Meta‐Analysis of Longitudinal Studies,” Journal of Affective Disorders 169 (2014): 61–75, 10.1016/j.jad.2014.08.006. 25154536

[11] E. Dragioti , J. Radua , M. Solmi , et al., “Global Population Attributable Fraction of Potentially Modifiable Risk Factors for Mental Disorders: A Meta‐Umbrella Systematic Review,” Molecular Psychiatry 27, no. 8 (2022): 3510–3519, 10.1038/s41380-022-01586-8. 35484237 PMC9708560

[12] R. T. Liu , “Childhood Adversities and Depression in Adulthood: Current Findings and Future Directions,” Clinical Psychology 24, no. 2 (2017): 140–153, 10.1111/cpsp.12190. 28924333 PMC5600284

[13] M. T. McKay , L. Kilmartin , A. Meagher , M. Cannon , C. Healy , and M. C. Clarke , “A Revised and Extended Systematic Review and Meta‐Analysis of the Relationship Between Childhood Adversity and Adult Psychiatric Disorder,” Journal of Psychiatric Research 156 (2022): 268–283, 10.1016/j.jpsychires.2022.10.015. 36274532

[14] V. J. Felitti , R. F. Anda , D. Nordenberg , et al., “Relationship of Childhood Abuse and Household Dysfunction to Many of the Leading Causes of Death in Adults: The Adverse Childhood Experiences (ACE) Study,” American Journal of Preventive Medicine 14, no. 4 (1998): 245–258, 10.1016/S0749-3797(98)00017-8. 9635069

[15] S. Madigan , A. A. Deneault , N. Racine , et al., “Adverse Childhood Experiences: A Meta‐Analysis of Prevalence and Moderators Among Half a Million Adults in 206 Studies,” World Psychiatry 22, no. 3 (2023): 463–471, 10.1002/wps.21122. 37713544 PMC10503911

[16] K. Petruccelli , J. Davis , and T. Berman , “Adverse Childhood Experiences and Associated Health Outcomes: A Systematic Review and Meta‐Analysis,” Child Abuse & Neglect 97 (2019): 104127, 10.1016/j.chiabu.2019.104127. 31454589

[17] X. Chang , X. Jiang , T. Mkandarwire , and M. Shen , “Associations Between Adverse Childhood Experiences and Health Outcomes in Adults Aged 18–59 Years,” PLoS One 14, no. 2 (2019): e0211850, 10.1371/journal.pone.0211850. 30730980 PMC6366931

[18] L. R. Grummitt , N. T. Kreski , S. G. Kim , J. Platt , K. M. Keyes , and K. A. McLaughlin , “Association of Childhood Adversity With Morbidity and Mortality in US Adults: A Systematic Review,” JAMA Paediatrics 175, no. 12 (2021): 1269–1278, 10.1001/jamapediatrics.2021.2320. PMC9059254 34605870

[19] E. T. C. Lippard and C. B. Nemeroff , “The Devastating Clinical Consequences of Child Abuse and Neglect: Increased Disease Vulnerability and Poor Treatment Response in Mood Disorders,” American Journal of Psychiatry 177, no. 1 (2020): 20–36, 10.1176/appi.ajp.2019.19010020. 31537091 PMC6939135

[20] M. Vallati , S. Cunningham , R. Mazurka , et al., “Childhood Maltreatment and the Clinical Characteristics of Major Depressive Disorder in Adolescence and Adulthood,” Journal of Abnormal Psychology (1965) 129, no. 5 (2020): 469–479, 10.1037/abn0000521. 32237880

[21] J. R. Baldwin , B. Wang , L. Karwatowska , et al., “Childhood Maltreatment and Mental Health Problems: A Systematic Review and Meta‐Analysis of Quasi‐Experimental Studies,” American Journal of Psychiatry 180, no. 2 (2023): 117–126, 10.1176/appi.ajp.20220174. 36628513 PMC7614155

[22] E. C. Braithwaite , R. M. O'Connor , M. Degli‐Esposti , N. Luke , and L. Bowes , “Modifiable Predictors of Depression Following Childhood Maltreatment: A Systematic Review and Meta‐Analysis,” Translational Psychiatry 7, no. 7 (2017): e1162, 10.1038/tp.2017.140. 28675390 PMC5538120

[23] E. A. G. Gallo , T. N. Munhoz , C. Loret de Mola , and J. Murray , “Gender Differences in the Effects of Childhood Maltreatment on Adult Depression and Anxiety: A Systematic Review and Meta‐Analysis,” Child Abuse & Neglect 79 (2018): 107–114, 10.1016/j.chiabu.2018.01.003. 29428878

[24] V. Warrier , A. S. F. Kwong , M. Luo , et al., “Gene–Environment Correlations and Causal Effects of Childhood Maltreatment on Physical and Mental Health: A Genetically Informed Approach,” Lancet Psychiatry 8, no. 5 (2021): 373–386, 10.1016/S2215-0366(20)30569-1. 33740410 PMC8055541

[25] M. H. Teicher , J. B. Gordon , and C. B. Nemeroff , “Recognizing the Importance of Childhood Maltreatment as a Critical Factor in Psychiatric Diagnoses, Treatment, Research, Prevention, and Education,” Molecular Psychiatry 27, no. 3 (2022): 1331–1338, 10.1038/s41380-021-01367-9. 34737457 PMC8567985

[26] A. Ceruso , M. Martínez‐Cengotitabengoa , A. Peters‐Corbett , M. J. Diaz‐Gutierrez , and M. Martínez‐Cengotitabengoa , “Alterations of the HPA Axis Observed in Patients With Major Depressive Disorder and Their Relation to Early Life Stress: A Systematic Review,” Neuropsychobiology 79, no. 6 (2020): 417–427, 10.1159/000506484. 32203965

[27] R. C. Silva , E. Maffioletti , M. Gennarelli , B. T. Baune , and A. Minelli , “Biological Correlates of Early Life Stressful Events in Major Depressive Disorder,” Psychoneuroendocrinology 125 (2021): 105103, 10.1016/j.psyneuen.2020.105103. 33360031

[28] B. Jones Harden , C. Simons , M. Johnson‐Motoyama , and R. Barth , “The Child Maltreatment Prevention Landscape: Where Are We Now, and Where Should We Go?,” Annals of the American Academy of Political and Social Science 692, no. 1 (2020): 97–118, 10.1177/0002716220978361.

[29] A. E. Austin , A. M. Lesak , and M. E. Shanahan , “Risk and Protective Factors for Child Maltreatment: A Review,” Current Epidemiology Reports 7, no. 4 (2020): 334–342, 10.1007/s40471-020-00252-3. 34141519 PMC8205446

[30] K. Maguire‐Jack , T. Negash , and K. J. Steinman , “Child Maltreatment Prevention Strategies and Needs,” Journal of Child and Family Studies 27, no. 11 (2018): 3572–3584, 10.1007/s10826-018-1179-0.

[31] United Nations , The 17 Goals (Department of Economic and Social Affairs, Sustainable Development, United Nations, 2024), https://sdgs.un.org/topics/violence‐against‐children.

[32] B. Mathews , H. J. Thomas , and J. G. Scott , “A New Era in Child Maltreatment Prevention: Call to Action,” Medical Journal of Australia 218, no. S6 (2023): S47–S51, 10.5694/mja2.51872. 37004187 PMC10952631

[33] M. Ramiro‐Gonzalez , D. Dobermann , D. Metilka , E. Aldridge , Y. Yon , and D. Sethi , “Child Maltreatment Prevention: A Content Analysis of European National Policies,” European Journal of Public Health 29, no. 1 (2019): 32–38, 10.1093/eurpub/cky176. 30184076 PMC6345150

[34] J. Nelson , A. Klumparendt , P. Doebler , and T. Ehring , “Childhood Maltreatment and Characteristics of Adult Depression: Meta‐Analysis,” British Journal of Psychiatry 210, no. 2 (2017): 96–104, 10.1192/bjp.bp.115.180752. 27908895

[35] E. Brehaut , D. Neupane , B. Levis , et al., “Depression Prevalence Using the HADS‐D Compared to SCID Major Depression Classification: An Individual Participant Data Meta‐Analysis,” Journal of Psychosomatic Research 139 (2020): 110256, 10.1016/j.jpsychores.2020.110256. 33069051

[36] L. Dang , L. Dong , and B. Mezuk , “Shades of Blue and Grey: A Comparison of the Centre for Epidemiologic Studies Depression Scale and the Composite International Diagnostic Interview for Assessment of Depression Syndrome in Later Life,” Gerontologist 60, no. 4 (2020): e242–e253, 10.1093/geront/gnz044. 31112598 PMC7228460

[37] A. L. Stuart , J. A. Pasco , F. N. Jacka , S. L. Brennan , M. Berk , and L. J. Williams , “Comparison of Self‐Report and Structured Clinical Interview in the Identification of Depression,” Comprehensive Psychiatry 55, no. 4 (2014): 866–869, 10.1016/j.comppsych.2013.12.019. 24467941

[38] M. J. Gardner , H. J. Thomas , and H. E. Erskine , “The Association Between Five Forms of Child Maltreatment and Depressive and Anxiety Disorders: A Systematic Review and Meta‐Analysis,” Child Abuse & Neglect 96 (2019): 104082, 10.1016/j.chiabu.2019.104082. 31374447

[39] M. Li , T. Gao , Y. Su , et al., “The Timing Effect of Childhood Maltreatment in Depression: A Systematic Review and Meta‐Analysis,” Trauma, Violence & Abuse 24, no. 4 (2023): 2560–2580, 10.1177/15248380221102558. 35608502

[40] M. D. De Bellis , K. B. Nooner , J. M. Scheid , and J. A. Cohen , “Depression in Maltreated Children and Adolescents,” Child and Adolescent Psychiatric Clinics of North America 28, no. 3 (2019): 289–302, 10.1016/j.chc.2019.02.002. 31076108

[41] G. Wells , B. Shea , D. O'Connell , et al., “The Newcastle‐Ottawa Scale (NOS) for Assessing the Quality of Nonrandomised Studies in Meta‐Analyses,” 2021, http://www.ohri.ca/programs/clinical_epidemiology/oxford.asp.

[42] J. P. T. Higgins , S. G. Thompson , J. J. Deeks , and D. G. Altman , “Measuring Inconsistency in Meta‐Analyses,” BMJ 327, no. 7414 (2003): 557–560, 10.1136/bmj.327.7414.557. 12958120 PMC192859

[43] S. Duval and R. L. Tweedie , “Trim and Fill: A Simple Funnel‐Plot‐Based Method of Testing and Adjusting for Publication Bias in Meta‐Analysis,” Biometrics 56 (2000): 455–463, 10.1111/j.0006-341X.2000.00455.x. 10877304

[44] T. O. Afifi , D. A. Brownridge , B. J. Cox , and J. Sareen , “Physical Punishment, Childhood Abuse and Psychiatric Disorders,” Child Abuse & Neglect 30, no. 10 (2006): 1093–1103, 10.1016/j.chiabu.2006.04.006. 17010436

[45] T. O. Afifi , J. Boman , W. Fleisher , and J. Sareen , “The Relationship Between Child Abuse, Parental Divorce, and Lifetime Mental Disorders and Suicidality in a Nationally Representative Adult Sample,” Child Abuse & Neglect 33, no. 3 (2009): 139–147, 10.1016/j.chiabu.2008.12.009. 19327835

[46] A. F. Al Shawi , Y. T. Sarhan , and M. A. Altaha , “Adverse Childhood Experiences and Their Relationship to Gender and Depression Among Young Adults in Iraq: A Cross‐Sectional Study,” BMC Public Health 19, no. 1 (2019): 1687, 10.1186/s12889-019-7957-9. 31842837 PMC6916082

[47] K. Amone‐P'Olak and N. K. Letswai , “The Relationship Between Adverse Childhood Experiences and Depression: A Cross‐Sectional Survey With University Students in Botswana,” South African Journal of Psychiatry 26 (2020): 1444, 10.4102/sajpsychiatry.v26i0.1444. PMC7669992 33240547

[48] I. Angelakis and P. Gooding , “Associations of Anxiety and Depression With Suicide Experiences in Individuals With and Without Childhood Trauma: The Role of Social Support,” Psychiatry Research 309 (2022): 114424, 10.1016/j.psychres.2022.114424. 35121339

[49] O. A. Babatunde , S. P. Ramkumar , S. A. Nguyen , et al., “Association Between Number of Adverse Childhood Experiences and Depression Among Older Adults Is Moderated by Race,” Preventive Medicine 181 (2024): 107921, 10.1016/j.ypmed.2024.107921. 38423302

[50] Ç. Berber Çelik and H. Odacı , “Does Child Abuse Have an Impact on Self‐Esteem, Depression, Anxiety and Stress Conditions of Individuals?,” International Journal of Social Psychiatry 66, no. 2 (2020): 171–178, 10.1177/0020764019894618. 31856622

[51] A. E. Bonomi , E. A. Cannon , M. L. Anderson , F. P. Rivara , and R. S. Thompson , “Association Between Self‐Reported Health and Physical and/or Sexual Abuse Experienced Before Age 18,” Child Abuse & Neglect 32, no. 7 (2008): 693–701, 10.1016/j.chiabu.2007.10.004. 18602692

[52] E. A. Cannon , A. E. Bonomi , M. L. Anderson , F. P. Rivara , and R. S. Thompson , “Adult Health and Relationship Outcomes Among Women With Abuse Experiences During Childhood,” Violence and Victims 25, no. 3 (2010): 291–305, 10.1891/0886-6708.25.3.291. 20565002

[53] C. Cavanaugh and T. Nelson , “A National Study of the Influence of Adverse Childhood Experiences on Depression Among Black Adults in the United States,” Journal of Affective Disorders 311 (2022): 523–529, 10.1016/j.jad.2022.05.112. 35605705

[54] D. P. Chapman , C. L. Whitfield , V. J. Felitti , S. R. Dube , V. J. Edwards , and R. F. Anda , “Adverse Childhood Experiences and the Risk of Depressive Disorders in Adulthood,” Journal of Affective Disorders 82, no. 2 (2004): 217–225, 10.1016/j.jad.2003.12.013. 15488250

[55] X. Chen , S. Zhang , G. Huang , et al., “Associations Between Child Maltreatment and Depressive Symptoms Among Chinese College Students: An Analysis of Sex Differences,” Frontiers in Psychiatry 12 (2021): 656646, 10.3389/fpsyt.2021.656646. 34305672 PMC8298832

[56] E. V. Cheong , C. Sinnott , D. Dahly , and P. M. Kearney , “Adverse Childhood Experiences (ACEs) and Later‐Life Depression: Perceived Social Support as a Potential Protective Factor,” BMJ Open 7, no. 9 (2017): e013228, 10.1136/bmjopen-2016-013228. PMC5588961 28864684

[57] H. C. Comijs , E. van Exel , R. C. van der Mast , A. Paauw , R. Oude Voshaar , and M. L. Stek , “Childhood Abuse in Late‐Life Depression,” Journal of Affective Disorders 147, no. 1 (2013): 241–246, 10.1016/j.jad.2012.11.010. 23196199

[58] M. Comtois‐Cabana , E. Barr , N. Provençal , and I. Ouellet‐Morin , “Association Between Child Maltreatment and Depressive Symptoms in Emerging Adulthood: The Mediating and Moderating Roles of DNA Methylation,” PLoS One 18, no. 1 (2023): e0280203, 10.1371/journal.pone.0280203. 36634080 PMC9836296

[59] E. Cong , Y. Li , C. Shao , et al., “Childhood Sexual Abuse and the Risk for Recurrent Major Depression in Chinese Women,” Psychological Medicine 42, no. 2 (2012): 409–417, 10.1017/S0033291711001462. 21835095 PMC3250087

[60] L. Cross , J. Warren‐Findlow , J. Bowling , C. L. Reeve , and L. M. Issel , “Reimagining the Measurement of Adverse Childhood Experiences: A Delphi Study to Develop ACE Dimension Items,” TPM: Testing, Psychometrics, Methodology in Applied Psychology 30, no. 3 (2023): 259–286.

[61] S. D. Easton , S. Roh , J. Kong , and Y.‐S. Lee , “Childhood Sexual Abuse and Depression Among American Indians in Adulthood,” Health & Social Work 44, no. 2 (2019): 95–103, 10.1093/hsw/hlz005. 30809642

[62] M. A. Ege , E. Messias , P. B. Thapa , and L. P. Krain , “Adverse Childhood Experiences and Geriatric Depression: Results From the 2010 BRFSS,” American Journal of Geriatric Psychiatry 23, no. 1 (2015): 110–114, 10.1016/j.jagp.2014.08.014. PMC4267899 25306195

[63] E. A. G. Gallo , C. L. De Mola , F. Wehrmeister , H. Gonçalves , C. Kieling , and J. Murray , “Childhood Maltreatment Preceding Depressive Disorder at Age 18 Years: A Prospective Brazilian Birth Cohort Study,” Journal of Affective Disorders 217 (2017): 218–224, 10.1016/j.jad.2017.03.065. 28431382 PMC5469396

[64] G. R. Ghassemi , S. Sadeghi , G. A. Asadollahi , A. R. Yousefy , and S. Mallik , “Early Experiences of Abuse and Current Depressive Disorders in Iranian Women,” Eastern Mediterranean Health Journal 16, no. 5 (2010): 498–504. 20799548

[65] K. S. Guerrero , M. K. Horton , V. Choudhary , et al., “Adverse Childhood Experiences in Early Life Increase the Odds of Depression Among Adults With Multiple Sclerosis,” Multiple Sclerosis Journal—Experimental, Translational and Clinical 9, no. 4 (2023): 20552173231202638, 10.1177/20552173231202638. 37808459 PMC10552460

[66] M. Y. Gürsoy and F. C. Mechmet , “Correlations Between Childhood Trauma and Depression, Anxiety, and Stress Levels in Nurses,” Archives of Psychiatric Nursing 45 (2023): 164–168, 10.1016/j.apnu.2023.06.018. 37544694

[67] A. M. Hatcher , A. Gibbs , R. Jewkes , R.‐S. McBride , D. Peacock , and N. Christofides , “Effect of Childhood Poverty and Trauma on Adult Depressive Symptoms Among Young Men in Peri‐Urban South African Settlements,” Journal of Adolescent Health 64, no. 1 (2019): 79–85, 10.1016/j.jadohealth.2018.07.026. 30327276

[68] Y. Hayashi , Y. Okamoto , K. Takagaki , et al., “Direct and Indirect Influences of Childhood Abuse on Depression Symptoms in Patients With Major Depressive Disorder,” BMC Psychiatry 15, no. 1 (2015): 244, 10.1186/s12888-015-0636-1. 26467656 PMC4604614

[69] J. G. F. M. Hovens , E. J. Giltay , J. E. Wiersma , P. Spinhoven , B. W. J. H. Penninx , and F. G. Zitman , “Impact of Childhood Life Events and Trauma on the Course of Depressive and Anxiety Disorders,” Acta Psychiatrica Scandinavica 126, no. 3 (2012): 198–207, 10.1111/j.1600-0447.2011.01828.x. 22268708

[70] K. S. Kendler , J. W. Kuhn , and C. A. Prescott , “Childhood Sexual Abuse, Stressful Life Events and Risk for Major Depression in Women,” Psychological Medicine 34, no. 8 (2004): 1475–1482, 10.1017/S003329170400265X. 15724878

[71] R. C. Kessler and W. J. Magee , “Childhood Family Violence and Adult Recurrent Depression,” Journal of Health and Social Behaviour 35, no. 1 (1994): 13–27. 8014427

[72] A. Khan , H. C. McCormack , E. A. Bolger , et al., “Childhood Maltreatment, Depression, and Suicidal Ideation: Critical Importance of Parental and Peer Emotional Abuse During Developmental Sensitive Periods in Males and Females,” Frontiers in Psychiatry 6 (2015): 42, 10.3389/fpsyt.2015.00042. 25870565 PMC4378368

[73] S.‐S. Kim , H. Jang , H. Y. Chang , Y. S. Park , and D.‐W. Lee , “Association Between Childhood Adversities and Adulthood Depressive Symptoms in South Korea: Results From a Nationally Representative Longitudinal Study,” BMJ Open 3, no. 7 (2013): e002680, 10.1136/bmjopen-2013-002680. PMC3717452 23878171

[74] A. R. King , “Childhood Adversity Links to Self‐Reported Mood, Anxiety, and Stress‐Related Disorders,” Journal of Affective Disorders 292 (2021): 623–632, 10.1016/j.jad.2021.05.112. 34153833

[75] S. Kisely , A. A. Abajobir , R. Mills , L. Strathearn , A. Clavarino , and J. M. Najman , “Child Maltreatment and Mental Health Problems in Adulthood: Birth Cohort Study,” British Journal of Psychiatry 213, no. 6 (2018): 698–703, 10.1192/bjp.2018.207. 30475193

[76] S. Kisely , L. Strathearn , and J. M. Najman , “Child Maltreatment and Mental Health Problems in 30‐Year‐Old Adults: A Birth Cohort Study,” Journal of Psychiatric Research 129 (2020): 111–117, 10.1016/j.jpsychires.2020.06.009. 32653613

[77] S. Kisely , L. Strathearn , R. Mills , and J. M. Najman , “A Comparison of the Psychological Outcomes of Self‐Reported and Agency‐Notified Child Abuse in a Population‐Based Birth Cohort at 30‐Year‐Follow‐Up,” Journal of Affective Disorders 280 (2021): 167–172, 10.1016/j.jad.2020.11.017. 33212408

[78] K. Korkeila , J. Korkeila , J. Vahtera , et al., “Childhood Adversities, Adult Risk Factors and Depressiveness: A Population Study,” Social Psychiatry and Psychiatric Epidemiology 40, no. 9 (2005): 700–706, 10.1007/s00127-005-0969-x. 16151596

[79] J. Korkeila , J. Vahtera , H. Nabi , et al., “Childhood Adversities, Adulthood Life Events and Depression,” Journal of Affective Disorders 127, no. 1 (2010): 130–138, 10.1016/j.jad.2010.04.031. 20569993

[80] M. A. Lara , L. Navarrete , L. Nieto , and H.‐N. Le , “Childhood Abuse Increases the Risk of Depressive and Anxiety Symptoms and History of Suicidal Behaviour in Mexican Pregnant Women,” Revista Brasileira de Psiquiatria 37, no. 3 (2015): 203–210, 10.1590/1516-4446-2014-1479. 26039189

[81] R. D. Lee and J. Chen , “Adverse Childhood Experiences, Mental Health, and Excessive Alcohol Use: Examination of Race/Ethnicity and Sex Differences,” Child Abuse & Neglect 69 (2017): 40–48, 10.1016/j.chiabu.2017.04.004. 28448813 PMC5896758

[82] K. LeMasters , L. M. Bates , E. O. Chung , et al., “Adverse Childhood Experiences and Depression Among Women in Rural Pakistan,” BMC Public Health 21, no. 1 (2021): 400, 10.1186/s12889-021-10409-4. 33632175 PMC7905421

[83] S. T. Lereya , W. E. Copeland , E. J. Costello , and D. Wolke , “Adult Mental Health Consequences of Peer Bullying and Maltreatment in Childhood: Two Cohorts in Two Countries,” Lancet Psychiatry 2, no. 6 (2015): 524–531, 10.1016/S2215-0366(15)00165-0. 26360448 PMC4580734

[84] J. Lian , K. M. Kiely , B. L. Callaghan , and K. J. Anstey , “Childhood Adversity Is Associated With Anxiety and Depression in Older Adults: A Cumulative Risk and Latent Class Analysis,” Journal of Affective Disorders 354 (2024): 181–190, 10.1016/j.jad.2024.03.016. 38484890

[85] L. Lin , B. Cao , W. Chen , J. Li , Y. Zhang , and V. Y. Guo , “Association of Childhood Threat and Deprivation With Depressive Symptoms and the Moderating Role of Current Economic Status Among Middle‐Aged and Older Adults in China,” Social Psychiatry and Psychiatric Epidemiology 58, no. 8 (2023): 1227–1236, 10.1007/s00127-022-02384-x. 36418644

[86] D. Loxton , P. M. Forder , D. Cavenagh , et al., “The Impact of Adverse Childhood Experiences on the Health and Health Behaviours of Young Australian Women,” Child Abuse & Neglect 111 (2021): 104771, 10.1016/j.chiabu.2020.104771. 33160649

[87] D. C. McFarland , C. Andreotti , K. Harris , J. Mandeli , A. Tiersten , and J. Holland , “Early Childhood Adversity and Its Associations With Anxiety, Depression, and Distress in Women With Breast Cancer,” Psychosomatics 57, no. 2 (2016): 174–184, 10.1016/j.psym.2015.11.008. 26876888 PMC5023013

[88] P. E. Mullen , J. L. Martin , J. C. Anderson , S. E. Romans , and G. P. Herbison , “The Long‐Term Impact of the Physical, Emotional, and Sexual Abuse of Children: A Community Study,” Child Abuse & Neglect 20, no. 1 (1996): 7–21, 10.1016/0145-2134(95)00112-3. 8640429

[89] M. Novelo , A. von Gunten , G. B. Gomes Jardim , L. Spanemberg , I. I. de Argimon , and E. L. Nogueira , “Effects of Childhood Multiple Maltreatment Experiences on Depression of Socioeconomic Disadvantaged Elderly in Brazil,” Child Abuse & Neglect 79 (2018): 350–357, 10.1016/j.chiabu.2018.02.013. 29522996

[90] I. Ouellet‐Morin , H. L. Fisher , M. York‐Smith , S. Fincham‐Campbell , T. E. Moffitt , and L. Arseneault , “Intimate Partner Violence and New‐Onset Depression: A Longitudinal Study of Women's Childhood and Adult Histories of Abuse,” Depression and Anxiety 32, no. 5 (2015): 316–324, 10.1002/da.22347. 25691224 PMC4418177

[91] A. D. Paradis , H. Z. Reinherz , R. M. Giaconia , W. R. Beardslee , K. Ward , and G. M. Fitzmaurice , “Long‐Term Impact of Family Arguments and Physical Violence on Adult Functioning at Age 30 Years: Findings From the Simmons Longitudinal Study,” Journal of the American Academy of Child and Adolescent Psychiatry 48, no. 3 (2009): 290–298, 10.1097/CHI.0b013e3181948fdd. 19182693

[92] A. Peng , X. Qiu , S. Ji , et al., “The Impact of Childhood Parental Loss on Risk for Depression and Anxiety in Adulthood: A Community‐Based Study in Southwest China,” Journal of Affective Disorders 298 (2022): 104–109, 10.1016/j.jad.2021.10.093. 34715182

[93] J. Petersen , A.‐C. Schulz , E. Brähler , C. Sachser , J. M. Fegert , and M. E. Beutel , “Childhood Maltreatment, Depression and Their Link to Adult Economic Burdens,” Frontiers in Psychiatry 13 (2022): 908422, 10.3389/fpsyt.2022.908422. 36072464 PMC9441673

[94] J. C. Poole , K. S. Dobson , and D. Pusch , “Childhood Adversity and Adult Depression: The Protective Role of Psychological Resilience,” Child Abuse & Neglect 64 (2017): 89–100, 10.1016/j.chiabu.2016.12.012. 28056359

[95] W. Rehan , J. Antfolk , A. Johansson , P. Jern , and P. Santtila , “Experiences of Severe Childhood Maltreatment, Depression, Anxiety and Alcohol Abuse Among Adults in Finland,” PLoS One 12, no. 5 (2017): e0177252, 10.1371/journal.pone.0177252. 28481912 PMC5421798

[96] H. Z. Reinherz , A. D. Paradis , R. M. Giaconia , C. K. Stashwick , and G. Fitzmaurice , “Childhood and Adolescent Predictors of Major Depression in the Transition to Adulthood,” American Journal of Psychiatry 160, no. 12 (2003): 2141–2147, 10.1176/appi.ajp.160.12.2141. 14638584

[97] R. A. Remigio‐Baker , D. K. Hayes , and F. Reyes‐Salvail , “Adverse Childhood Events and Current Depressive Symptoms Among Women in Hawaii: 2010 BRFSS, Hawaii,” Maternal and Child Health Journal 18, no. 10 (2014): 2300–2308, 10.1007/s10995-013-1374-y. 24178156

[98] N. Roland , C. Leon , E. du Roscoat , H. Panjo , M.‐J. Saurel‐Cubizolles , and V. Ringa , “Witnessing Interparental Violence in Childhood and Symptoms of Depression in Adulthood: Data From the 2017 French Health Barometer,” Family Practice 38, no. 3 (2021): 306–312, 10.1093/fampra/cmaa127. 33251547

[99] I. A. Rubino , R. C. Nanni , D. M. Pozzi , and A. Siracusano , “Early Adverse Experiences in Schizophrenia and Unipolar Depression,” Journal of Nervous and Mental Disease 197, no. 1 (2009): 65–68, 10.1097/NMD.0b013e3181925342. 19155813

[100] S. Rudenstine , G. Cohen , M. Prescott , et al., “Adverse Childhood Events and the Risk for New‐Onset Depression and Post‐Traumatic Stress Disorder Among US National Guard Soldiers,” Military Medicine 180, no. 9 (2015): 972–978, 10.7205/MILMED-D-14-00626. 26327549

[101] D. Russell , K. W. Springer , and E. A. Greenfield , “Witnessing Domestic Abuse in Childhood as an Independent Risk Factor for Depressive Symptoms in Young Adulthood,” Child Abuse & Neglect 34, no. 6 (2010): 448–453, 10.1016/j.chiabu.2009.10.004. 20409587 PMC2872053

[102] A. Saleh , G. G. Potter , D. R. McQuoid , et al., “Effects of Early Life Stress on Depression, Cognitive Performance and Brain Morphology,” Psychological Medicine 47, no. 1 (2017): 171–181, 10.1017/S0033291716002403. 27682320 PMC5195852

[103] E. N. Satinsky , B. Kakuhikire , C. Baguma , et al., “Adverse Childhood Experiences, Adult Depression, and Suicidal Ideation in Rural Uganda: A Cross‐Sectional, Population‐Based Study,” PLoS Medicine 18, no. 5 (2021): e1003642, 10.1371/journal.pmed.1003642. 33979329 PMC8153443

[104] E. A. Schilling , R. H. Aseltine , and S. Gore , “Adverse Childhood Experiences and Mental Health in Young Adults: A Longitudinal Survey,” BMC Public Health 7, no. 1 (2007): 30, 10.1186/1471-2458-7-30. 17343754 PMC1832182

[105] K. M. Scott , K. A. McLaughlin , D. A. R. Smith , and P. M. Ellis , “Childhood Maltreatment and DSM‐IV Adult Mental Disorders: Comparison of Prospective and Retrospective Findings,” British Journal of Psychiatry 200, no. 6 (2012): 469–475, 10.1192/bjp.bp.111.103267. PMC3365274 22661679

[106] J. G. Scott , E. Malacova , B. Mathews , et al., “The Association Between Child Maltreatment and Mental Disorders in the Australian Child Maltreatment Study,” Medical Journal of Australia 218, no. S6 (2023): S26–S33, 10.5694/mja2.51870. 37004186 PMC10952950

[107] L. Shanahan , W. E. Copeland , E. J. Costello , and A. Angold , “Child‐, Adolescent‐ and Young Adult‐Onset Depressions: Differential Risk Factors in Development?,” Psychological Medicine 41, no. 11 (2011): 2265–2274, 10.1017/S0033291711000675. 21557889 PMC3181285

[108] T. L. Taillieu , D. A. Brownridge , J. Sareen , and T. O. Afifi , “Childhood Emotional Maltreatment and Mental Disorders: Results From a Nationally Representative Adult Sample From the United States,” Child Abuse & Neglect 59 (2016): 1–12, 10.1016/j.chiabu.2016.07.005. 27490515

[109] S. Telfar , G. F. H. McLeod , B. Dhakal , et al., “Child Abuse and Neglect and Mental Health Outcomes in Adulthood by Ethnicity: Findings From a 40‐Year Longitudinal Study in New Zealand/Aotearoa,” Child Abuse & Neglect 145 (2023): 106444, 10.1016/j.chiabu.2023.106444. 37703676

[110] M. Tracy , M. Salo , N. Slopen , T. Udo , and A. A. Appleton , “Trajectories of Childhood Adversity and the Risk of Depression in Young Adulthood: Results From the Avon Longitudinal Study of Parents and Children,” Depression and Anxiety 36, no. 7 (2019): 596–606, 10.1002/da.22887. 30884010 PMC6602824

[111] E. Van Overloop , C. Arms‐Chavez , R. N. Carol , and S. G. LoBello , “Effects of Adverse Childhood Experiences and Chronic Health Conditions on Current Depression,” Community Mental Health Journal 59, no. 6 (2023): 1208–1216, 10.1007/s10597-023-01103-3. 36840804

[112] R. Waite and P. A. Shewokis , “Childhood Trauma and Adult Self‐Reported Depression,” ABNF Journal 23, no. 1 (2012): 8–13. 23387107

[113] A. Wajid , S. V. van Zanten , M. K. Mughal , et al., “Adversity in Childhood and Depression in Pregnancy,” Archives of Women's Mental Health 23, no. 2 (2020): 169–180, 10.1007/s00737-019-00966-4. 31016472

[114] R. C. Whitaker , T. Dearth‐Wesley , A. N. Herman , et al., “The Interaction of Adverse Childhood Experiences and Gender as Risk Factors for Depression and Anxiety Disorders in US Adults: A Cross‐Sectional Study,” BMC Public Health 21, no. 1 (2021): 2078, 10.1186/s12889-021-12058-z. 34772386 PMC8590371

[115] L. A. Wise , S. Zierler , N. Krieger , and B. L. Harlow , “Adult Onset of Major Depressive Disorder in Relation to Early Life Violent Victimisation: A Case–Control Study,” Lancet 358, no. 9285 (2001): 881–887, 10.1016/S0140-6736(01)06072-X. 11567704

[116] X. Xiang and X. Wang , “Childhood Adversity and Major Depression in Later Life: A Competing‐Risks Regression Analysis,” International Journal of Geriatric Psychiatry 36, no. 1 (2021): 215–223, 10.1002/gps.5417. 32869351

[117] R. Ye , J. Li , Y. Du , H. Wang , and J. Gu , “Experience of Childhood Sexual Violence and Its Associations With Depressive Symptoms Among University Students in Guangdong, China,” Journal of Affective Disorders 321 (2023): 234–241, 10.1016/j.jad.2022.10.039. 36336165

[118] R. Yin , Y. Yang , L. Tang , Y. Chang , and F. Zhang , “Childhood Abuse and Association With Adult Depressive Symptoms Among People With Cardiovascular Disease,” Frontiers in Public Health 11 (2023): 1179384, 10.3389/fpubh.2023.1179384. 37333526 PMC10273208

[119] T. Zhang , L. Kan , C. Jin , and W. Shi , “Adverse Childhood Experiences and Their Impacts on Subsequent Depression and Cognitive Impairment in Chinese Adults: A Nationwide Multi‐Centre Study,” Journal of Affective Disorders 323 (2023): 884–892, 10.1016/j.jad.2022.12.058. 36566934

[120] World Health Organization , Adverse Childhood Experiences International Questionnaire (ACE‐IQ) – Rationale for ACE‐IQ (WHO, 2012).

[121] American Psychiatric Association , Diagnostic and Statistical Manual of Mental Disorders, 5th ed. (APA, 2022), 10.1176/appi.books.9780890425787.

[122] A. T. Beck , R. A. Steer , and M. G. Carbin , “Psychometric Properties of the Beck Depression Inventory: Twenty‐Five Years of Evaluation,” Clinical Psychology Review 8, no. 1 (1988): 77–100, 10.1016/0272-7358(88)90050-5.

[123] L. R. Derogatis and N. Melisaratos , “The Brief Symptom Inventory: An Introductory Report,” Psychological Medicine 13, no. 3 (1983): 595–605, 10.1017/S0033291700048017. 6622612

[124] A. Angold and E. J. Costello , “The Child and Adolescent Psychiatric Assessment (CAPA),” Journal of the American Academy of Child and Adolescent Psychiatry 39 (2000): 39–48, 10.1097/00004583-200001000-00015. 10638066

[125] B. Sanders and E. Becker‐Lausen , “The Measurement of Psychological Maltreatment: Early Data on the Child Abuse and Trauma Scale,” Child Abuse & Neglect 19 (1995): 315–323. 9278731 10.1016/s0145-2134(94)00131-6

[126] D. J. Higgins and M. P. McCabe , “The Development of the Comprehensive Child Maltreatment Scale,” Journal of Family Studies 7, no. 1 (2001): 7–28, 10.5172/jfs.7.1.7.

[127] A. Bifulco , O. Bernazzani , P. M. Moran , and C. Jacobs , “The Childhood Experience of Care and Abuse Questionnaire (CECA.Q): Validation in a Community Series,” British Journal of Clinical Psychology 44, no. 4 (2005): 563–581, 10.1348/014466505X35344. 16368034

[128] P. M. Lewinsohn , J. R. Seeley , N. B. Allen , and R. E. Roberts , “Centre for Epidemiologic Studies Depression Scale (CES‐D) as a Screening Instrument for Depression Among Community‐Residing Older Adults,” Psychology and Aging 12, no. 2 (1997): 277–287, 10.1037/0882-7974.12.2.277. 9189988

[129] H.‐U. Wittchen , “Reliability and Validity Studies of the WHO‐Composite International Diagnostic Interview (CIDI): A Critical Review,” Journal of Psychiatric Research 28, no. 1 (1994): 57–84, 10.1016/0022-3956(94)90036-1. 8064641

[130] R. C. Kessler , G. Andrews , D. Mroczek , B. Ustun , and H. U. Wittchen , “The World Health Organization Composite International Diagnostic Interview Short‐Form (CIDI‐SF),” International Journal of Methods in Psychiatric Research 7, no. 4 (1998): 171–185. 10.1002/mpr.168 PMC6878592 15297906

[131] G. Lewis , A. J. Pelosi , R. Araya , and G. Dunn , “Measuring Psychiatric Disorder in the Community: A Standardized Assessment for Use by Lay Interviewers,” Psychological Medicine 22, no. 2 (1992): 465–486, 10.1017/s0033291700030415. 1615114

[132] D. P. Bernstein , L. Fink , L. Handelsman , et al., “Initial Reliability and Validity of a New Retrospective Measure of Child Abuse and Neglect,” American Journal of Psychiatry 151, no. 8 (1994): 1132–1136, 10.1176/ajp.151.8.1132. 8037246

[133] D. P. Bernstein , J. A. Stein , M. D. Newcomb , et al., “Development and Validation of a Brief Screening Version of the Childhood Trauma Questionnaire,” Child Abuse & Neglect 27, no. 2 (2003): 169–190, 10.1016/s0145-2134(02)00541-0. 12615092

[134] M. A. Straus , “Measuring Intrafamily Conflict and Violence: The Conflict Tactics (CT) Scales,” Journal of Marriage and Family 41, no. 1 (1979): 75–88, 10.2307/351733.

[135] P. F. Lovibond and S. H. Lovibond , “The Structure of Negative Emotional States: Comparison of the Depression Anxiety Stress Scales (DASS) With the Beck Depression and Anxiety Inventories,” Behaviour Research and Therapy 33 (1995): 335–343, 10.1016/0005-7967(94)00075-u. 7726811

[136] T. Sheeran and M. Zimmerman , “Case Identification of Depression With Self‐Report Questionnaires,” Psychiatry Research 109, no. 1 (2002): 51–59, 10.1016/S0165-1781(01)00364-X. 11850051

[137] J. L. Cox , J. M. Holden , and R. Sagovsky , “Detection of Postnatal Depression. Development of the 10‐item Edinburgh Postnatal Depression Scale,” The British Journal of Psychiatry: The Journal of Mental Science 150 (1987): 782–786, 10.1192/bjp.150.6.782. 3651732

[138] D. Goldberg , K. Bridges , P. Duncan‐Jones , and D. Grayson , “Detecting Anxiety and Depression in General Medical Settings,” British Medical Journal 297, no. 6653 (1988): 897–899, 10.1136/bmj.297.6653.897. 3140969 PMC1834427

[139] A. S. Zigmond and R. P. Snaith , “The Hospital Anxiety and Depression Scale,” Acta Psychiatrica Scandinavica 67, no. 6 (1983): 361–370, 10.1111/j.1600-0447.1983.tb09716.x. 6880820

[140] L. R. Derogatis , R. S. Lipman , and L. Covi , “SCL‐90: An Outpatient Psychiatric Rating Scale—Preliminary Report,” Psychopharmacology Bulletin 9, no. 1 (1973): 13–28. 4682398

[141] A. J. Rush , C. M. Gullion , M. R. Basco , R. B. Jarrett , and M. H. Trivedi , “The Inventory of Depressive Symptomatology (IDS): Psychometric Properties,” Psychological Medicine 26, no. 3 (1996): 477–486, 10.1017/s0033291700035558. 8733206

[142] D. Finkelhor , S. L. Hamby , R. Ormrod , and H. Turner , “The Juvenile Victimization Questionnaire: Reliability, Validity, and National Norms,” Child Abuse & Neglect 29, no. 4 (2005): 383–412, 10.1016/j.chiabu.2004.11.001. 15917079

[143] R. C. Kessler , G. Andrews , L. J. Colpe , et al., “Short Screening Scales to Monitor Population Prevalences and Trends in Non‐Specific Psychological Distress,” Psychological Medicine 32, no. 6 (2002): 959–976, 10.1017/s0033291702006074. 12214795

[144] M. H. Teicher and A. Parigger , “The ‘Maltreatment and Abuse Chronology of Exposure’ (MACE) Scale for the Retrospective Assessment of Abuse and Neglect During Development,” PLoS One 10, no. 2 (2015): e0117423, 10.1371/journal.pone.0117423. 25714856 PMC4340880

[145] S. A. Montgomery and M. Asberg , “A New Depression Scale Designed to Be Sensitive to Change,” British Journal of Psychiatry 134, no. 4 (1979): 382–389, 10.1192/bjp.134.4.382. 444788

[146] J. McFarlane , B. Parker , K. Soeken , and L. Bullock , “Assessing for Abuse During Pregnancy: Severity and Frequency of Injuries and Associated Entry Into Prenatal Care,” JAMA 267, no. 23 (1992): 3176–3178, 10.1001/jama.1992.03480230068030. 1593739

[147] B. Löwe , I. Wahl , M. Rose , et al., “A 4‐Item Measure of Depression and Anxiety: Validation and Standardization of the Patient Health Questionnaire‐4 (PHQ‐4) in the General Population,” Journal of Affective Disorders 122, no. 1 (2010): 86–95, 10.1016/j.jad.2009.06.019. 19616305

[148] K. Kroenke , T. W. Strine , R. L. Spitzer , J. B. W. Williams , J. T. Berry , and A. H. Mokdad , “The PHQ‐8 as a Measure of Current Depression in the General Population,” Journal of Affective Disorders 114, no. 1 (2009): 163–173, 10.1016/j.jad.2008.06.026. 18752852

[149] K. Kroenke and R. Spitzer , “The PHQ‐9: A New Depression Diagnostic and Severity Measure,” Psychiatric Annals 32, no. 9 (2002): 509–515, 10.3928/0048-5713-20020901-06.

[150] J. Smith , G. Fisher , L. Ryan , P. Clarke , J. House , and D. Weir , Psychosocial and Lifestyle Questionnaire (Survey Research Centre, Institute for Social Research, 2013).

[151] J. K. Wing , J. M. Nixon , S. A. Mann , and J. P. Leff , “Reliability of the PSE (Ninth Edition) Used in a Population Study,” Psychological Medicine 7, no. 3 (1977): 505–516, 10.1017/s0033291700004487. 905467

[152] S. E. Taylor , J. S. Lerner , R. M. Sage , B. J. Lehman , and T. E. Seeman , “Early Environment, Emotions, Responses to Stress, and Health,” Journal of Personality 72, no. 6 (2004): 1365–1394, 10.1111/j.1467-6494.2004.00300.x. 15509286

[153] W. W. K. Zung , “The Depression Status Inventory: An Adjunct to the Self‐Rating Depression Scale,” Journal of Clinical Psychology 28, no. 4 (1972): 539–543, 10.1002/1097-4679(197210)28:4<539::AID-JCLP2270280427>3.0.CO;2-S. 5080837

[154] A. Angold , E. J. Costello , S. C. Messer , and A. Pickles , “Development of a Short Questionnaire for Use in Epidemiological Studies of Depression in Children and Adolescents,” International Journal of Methods in Psychiatric Research 5, no. 4 (1995): 237–249.

[155] J. B. W. Williams , M. Link , N. E. Rosenthal , and M. Terman , Structured Interview Guide for the Hamilton Depression Rating Scale—Seasonal Affective Disorder Version (SIGH‐SAD) (New York Psychiatric Institute, 1988).

[156] A. Angold , A. Cox , M. Prendergast , et al., The Young Adult Psychiatric Assessment (YAPA) (Duke University Medical Centre, 1999).

[157] M. R. Schmidt , A. J. Narayan , V. M. Atzl , L. M. Rivera , and A. F. Lieberman , “Childhood Maltreatment on the Adverse Childhood Experiences (ACEs) Scale Versus the Childhood Trauma Questionnaire (CTQ) in a Perinatal Sample,” Journal of Aggression, Maltreatment & Trauma 29, no. 1 (2020): 38–56, 10.1080/10926771.2018.1524806.

[158] K. A. McLaughlin , M. A. Sheridan , K. L. Humphreys , J. Belsky , and B. J. Ellis , “The Value of Dimensional Models of Early Experience: Thinking Clearly About Concepts and Categories,” Perspectives on Psychological Science 16, no. 6 (2021): 1463–1472, 10.1177/1745691621992346. 34491864 PMC8563369

[159] T. D. Cosco , M. Prina , B. Stubbs , and Y. T. Wu , “Reliability and Validity of the Centre for Epidemiologic Studies Depression Scale in a Population‐Based Cohort of Middle‐Aged U.S. Adults,” Journal of Nursing Measurement 25, no. 3 (2017): 476–485, 10.1891/1061-3749.25.3.476. 29268830

[160] M. Mohebbi , V. Nguyen , J. J. McNeil , et al., “Psychometric Properties of a Short Form of the Centre for Epidemiologic Studies Depression (CES‐D‐10) Scale for Screening Depressive Symptoms in Healthy Community Dwelling Older Adults,” General Hospital Psychiatry 51 (2018): 118–125, 10.1016/j.genhosppsych.2017.08.002. 28890280 PMC6178798

[161] C. A. Hitchon , L. Zhang , C. A. Peschken , et al., “Validity and Reliability of Screening Measures for Depression and Anxiety Disorders in Rheumatoid Arthritis,” Arthritis Care & Research 2010 72, no. 8 (2020): 1130–1139, 10.1002/acr.24011. 31199570 PMC7496677

[162] R. A. Marrie , L. Zhang , L. M. Lix , et al., “The Validity and Reliability of Screening Measures for Depression and Anxiety Disorders in Multiple Sclerosis,” Multiple Sclerosis and Related Disorders 20 (2018): 9–15, 10.1016/j.msard.2017.12.007. 29274564

[163] C. Gayer‐Anderson , U. Reininghaus , I. Paetzold , et al., “A Comparison Between Self‐Report and Interviewer‐Rated Retrospective Reports of Childhood Abuse Among Individuals With First‐Episode Psychosis and Population‐Based Controls,” Journal of Psychiatric Research 123 (2020): 145–150, 10.1016/j.jpsychires.2020.02.002. 32065950 PMC7054833

