# Supplementary material for: A Systematic Review and Meta‐Analysis of the Association Between Childhood Maltreatment and Adult Depression
Source: Acta Psychiatr Scand. 2025 Mar 3;151(5):572–99. doi: 10.1111/acps.13794 (PMC11962359; doi:10.1111/acps.13794)
Supplement: Supplementary file 1 — Data S1. Supporting Information. [file ACPS-151-572-s001.docx]

Supplementary Materials

**Supplementary Table 1**

*Quality Analysis of Identified Studies Using the Newcastle-Ottawa Quality Assessment Scale*

| **Authors** | **Year** | **Sample Size**^1^ | **Study Design**^2^ | **Appropriately Conducted**^3^ | **Representativeness of the Exposed Group**^4^ | **Selection of the Control Group**^5^ | **Ascertainment of Exposure**^6^ | **Test for childhood maltreatment** ^7^ | **Assessment of depression** ^8^ | **Appropriate statistical analysis** ^9^ | **Appropriate methods to control confounding** | **Analysis results were appropriately interpreted** ^11^ | **Result** |
| --- | --- | --- | --- | --- | --- | --- | --- | --- | --- | --- | --- | --- | --- |
| Afifi et al. | 2006 | 1 | 1 | 1 | 1 | 1 | 0 | 0 | 1 | 1 | 0 | 1 | 8 |
| Afifi et al. | 2009 | 1 | 1 | 1 | 1 | 1 | 0 | 0 | 1 | 1 | 0 | 1 | 8 |
| Al Shawi et al. | 2019 | 0 | 1 | 1 | 0 | 0 | 0 | 0 | 0 | 1 | 0 | 1 | 4 |
| Amone-P’Olak & Letswai | 2020 | 1 | 1 | 1 | 0 | 0 | 0 | 0 | 0 | 1 | 0 | 1 | 5 |
| Angelakis & Gooding | 2022 | 1 | 1 | 1 | 1 | 1 | 0 | 0 | 0 | 0 | 0 | 1 | 6 |
| Babatunde et al. | 2024 | 1 | 1 | 1 | 0 | 0 | 0 | 0 | 0 | 1 | 1 | 1 | 6 |
| Berber Celic & Odaci | 2019 | 1 | 1 | 1 | 0 | 0 | 0 | 0 | 0 | 0 | 0 | 1 | 4 |
| Bonomi et al. | 2008 | 1 | 1 | 1 | 0 | 0 | 0 | 0 | 0 | 1 | 1 | 1 | 6 |
| Cannon et al. | 2010 | 1 | 1 | 1 | 0 | 0 | 0 | 0 | 0 | 1 | 0 | 1 | 5 |
| Cavanaugh & Nelson | 2022 | 1 | 1 | 1 | 0 | 0 | 0 | 0 | 1 | 1 | 1 | 1 | 7 |
| Chang et al. | 2019 | 1 | 1 | 1 | 1 | 1 | 0 | 0 | 0 | 1 | 1 | 1 | 8 |
| Chapman et al. | 2004 | 1 | 1 | 1 | 0 | 0 | 0 | 0 | 1 | 1 | 1 | 1 | 7 |
| Chen et al. | 2021 | 1 | 1 | 1 | 0 | 0 | 0 | 0 | 0 | 1 | 1 | 1 | 7 |
| Cheong et al. | 2017 | 1 | 1 | 1 | 0 | 0 | 0 | 0 | 0 | 1 | 1 | 1 | 6 |
| Comijs et al. | 2013 | 1 | 1 | 1 | 1 | 1 | 0 | 0 | 1 | 1 | 1 | 1 | 9 |
| Comtois-Cabana et al. | 2023 | 0 | 1 | 0 | 1 | 1 | 0 | 0 | 0 | 0 | 1 | 1 | 5 |
| Cong et al. | 2012 | 1 | 1 | 1 | 0 | 0 | 0 | 0 | 1 | 1 | 1 | 1 | 5 |
| Cross et al. | 2023 | 1 | 1 | 1 | 0 | 0 | 0 | 0 | 0 | 1 | 0 | 1 | 5 |
| Easton et al. | 2019 | 1 | 1 | 1 | 0 | 0 | 0 | 0 | 0 | 0 | 0 | 1 | 4 |
| Ege et al. | 2015 | 1 | 1 | 1 | 1 | 1 | 0 | 0 | 0 | 1 | 0 | 1 | 7 |
| Gallo et al. | 2017 | 1 | 1 | 1 | 0 | 0 | 1 | 0 | 1 | 1 | 1 | 1 | 8 |
| Ghassemi et al. | 2010 | 1 | 1 | 1 | 0 | 0 | 0 | 0 | 0 | 1 | 1 | 1 | 6 |
| Guerrero et al. | 2023 | 1 | 1 | 1 | 0 | 0 | 0 | 0 | 0 | 1 | 0 | 1 | 5 |
| Gursoy et al. | 2023 | 1 | 1 | 1 | 0 | 0 | 0 | 0 | 0 | 0 | 1 | 1 | 5 |
| Hatcher et al. | 2019 | 1 | 1 | 1 | 0 | 0 | 0 | 0 | 0 | 1 | 1 | 1 | 6 |
| Hayashi et al. | 2015 | 0 | 0 | 1 | 1 | 0 | 0 | 0 | 0 | 0 | 1 | 1 | 4 |
| Hovens et al. | 2012 | 1 | 1 | 1 | 1 | 1 | 0 | 0 | 1 | 1 | 1 | 1 | 9 |
| Kendler et al. | 2004 | 1 | 1 | 1 | 0 | 0 | 0 | 0 | 1 | 1 | 0 | 1 | 6 |
| Kessler & Magee | 1994 | 1 | 1 | 1 | 1 | 1 | 0 | 0 | 1 | 1 | 1 | 1 | 9 |
| Khan et al. | 2015 | 1 | 1 | 1 | 1 | 1 | 0 | 0 | 0 | 1 | 1 | 1 | 8 |
| Kim et al. | 2013 | 1 | 1 | 1 | 1 | 1 | 0 | 0 | 0 | 1 | 1 | 1 | 8 |
| King | 2021 | 1 | 1 | 1 | 0 | 0 | 0 | 0 | 0 | 0 | 0 | 1 | 8 |
| Kisley et al. | 2018 | 1 | 1 | 1 | 0 | 0 | 1 | 1 | 0 | 1 | 1 | 1 | 8 |
| Kisley et al. | 2020 | 1 | 1 | 1 | 0 | 0 | 1 | 1 | 1 | 1 | 1 | 1 | 9 |
| Kisley et al. | 2021 | 1 | 1 | 1 | 0 | 0 | 1 | 0 | 1 | 1 | 1 | 1 | 8 |
| Korkeila et al. | 2005 | 1 | 1 | 1 | 1 | 1 | 0 | 0 | 0 | 1 | 1 | 1 | 8 |
| Korkeila et al. | 2010 | 1 | 1 | 1 | 1 | 1 | 0 | 0 | 0 | 1 | 1 | 1 | 8 |
| Lara et al. | 2015 | 1 | 1 | 1 | 0 | 0 | 0 | 0 | 0 | 1 | 0 | 1 | 5 |
| Lee & Chen | 2017 | 1 | 1 | 1 | 1 | 1 | 0 | 0 | 0 | 1 | 0 | 1 | 7 |
| LeMasters et al. | 2021 | 1 | 1 | 1 | 0 | 0 | 1 | 0 | 1 | 1 | 0 | 1 | 7 |
| Lereya et al. | 2015 | 1 | 1 | 1 | 0 | 0 | 1 | 0 | 0 | 1 | 1 | 1 | 6 |
| Lian et al. | 2024 | 1 | 1 | 1 | 1 | 1 | 0 | 0 | 0 | 0 | 0 | 1 | 6 |
| Lin et al. | 2023 | 1 | 1 | 1 | 1 | 1 | 0 | 0 | 0 | 1 | 1 | 1 | 7 |
| Loxton et al. | 2021 | 1 | 1 | 1 | 0 | 0 | 1 | 0 | 0 | 1 | 0 | 1 | 6 |
| McFarland et al. | 2016 | 0 | 0 | 1 | 1 | 0 | 0 | 0 | 0 | 1 | 0 | 1 | 4 |
| Mullen et al. | 1996 | 1 | 1 | 1 | 1 | 1 | 0 | 0 | 0 | 1 | 1 | 1 | 8 |
| Novelo et al. | 2018 | 1 | 1 | 1 | 1 | 1 | 0 | 0 | 0 | 1 | 0 | 1 | 7 |
| Ouellet-Morin et al. | 2015 | 1 | 1 | 1 | 0 | 0 | 0 | 0 | 1 | 1 | 1 | 1 | 7 |
| Paradis et al. | 2009 | 1 | 1 | 1 | 1 | 1 | 0 | 0 | 1 | 1 | 1 | 1 | 8 |
| Peng et al. | 2022 | 1 | 1 | 1 | 1 | 1 | 0 | 0 | 0 | 1 | 0 | 1 | 7 |
| Petersen et al. | 2022 | 1 | 1 | 1 | 1 | 1 | 0 | 0 | 0 | 1 | 0 | 1 | 7 |
| Poole & Dobson | 2017 | 1 | 1 | 1 | 0 | 0 | 0 | 0 | 0 | 1 | 1 | 1 | 6 |
| Rehan et al. | 2017 | 1 | 1 | 1 | 1 | 1 | 0 | 0 | 0 | 1 | 0 | 1 | 7 |
| Reinherz et al. | 2003 | 1 | 1 | 1 | 1 | 1 | 0 | 0 | 1 | 1 | 1 | 1 | 9 |
| Remigio-Baker et al. | 2014 | 1 | 1 | 1 | 1 | 1 | 0 | 0 | 0 | 1 | 1 | 1 | 8 |
| Roland et al. | 2021 | 1 | 1 | 1 | 1 | 1 | 0 | 0 | 1 | 1 | 1 | 1 | 9 |
| Rubino et al. | 2009 | 1 | 1 | 1 | 0 | 0 | 0 | 0 | 1 | 1 | 0 | 1 | 6 |
| Rudenstein et al. | 2015 | 1 | 1 | 1 | 0 | 0 | 0 | 0 | 0 | 1 | 0 | 1 | 5 |
| Russell et al. | 2010 | 1 | 1 | 1 | 1 | 1 | 0 | 0 | 0 | 0 | 0 | 1 | 5 |
| Saleh et al. | 2017 | 0 | 0 | 1 | 1 | 0 | 0 | 0 | 1 | 0 | 0 | 1 | 4 |
| Satinsky et al. | 2021 | 1 | 1 | 1 | 1 | 1 | 0 | 0 | 0 | 1 | 1 | 1 | 8 |
| Schilling et al. | 2007 | 1 | 1 | 1 | 1 | 1 | 0 | 0 | 0 | 0 | 0 | 1 | 9 |
| Scott et al. | 2012 | 1 | 1 | 1 | 1 | 1 | 0 | 0 | 1 | 1 | 1 | 1 | 9 |
| Scott et al. | 2023 | 1 | 1 | 1 | 1 | 1 | 0 | 0 | 1 | 1 | 1 | 1 | 9 |
| Shanahan et al. | 2011 | 1 | 1 | 1 | 1 | 1 | 0 | 0 | 0 | 1 | 0 | 1 | 7 |
| Taillieu et al. | 2016 | 1 | 1 | 1 | 1 | 1 | 0 | 0 | 1 | 1 | 1 | 1 | 7 |
| Telfar et al. | 2023 | 1 | 1 | 1 | 0 | 0 | 0 | 0 | 1 | 1 | 1 | 1 | 7 |
| Tracy et al. | 2019 | 1 | 1 | 1 | 1 | 1 | 0 | 0 | 1 | 1 | 0 | 1 | 8 |
| Van Overloop et al. | 2023 | 1 | 1 | 1 | 1 | 1 | 0 | 0 | 0 | 1 | 1 | 1 | 8 |
| Waite & Shewokis | 2012 | 1 | 1 | 1 | 0 | 0 | 0 | 0 | 0 | 1 | 0 | 1 | 5 |
| Wajid et al. | 2020 | 1 | 1 | 1 | 0 | 0 | 0 | 0 | 1 | 1 | 1 | 1 | 6 |
| Whitaker et al. | 2021 | 1 | 1 | 1 | 1 | 1 | 0 | 0 | 1 | 1 | 0 | 1 | 8 |
| Wise et al. | 2001 | 1 | 1 | 1 | 1 | 1 | 0 | 0 | 1 | 1 | 1 | 1 | 9 |
| Xiang & Wang | 2021 | 1 | 1 | 1 | 0 | 0 | 0 | 0 | 1 | 1 | 1 | 1 | 7 |
| Ye et al. | 2023 | 1 | 1 | 1 | 0 | 0 | 0 | 0 | 0 | 1 | 1 | 1 | 6 |
| Yin et al. | 2023 | 1 | 1 | 1 | 0 | 0 | 0 | 0 | 0 | 1 | 1 | 1 | 6 |
| Zhang et al. | 2023 | 1 | 1 | 1 | 1 | 1 | 0 | 0 | 0 | 1 | 1 | 1 | 8 |

*Note:* ^1^Was the sample size large enough to provide enough power for analysis: Yes = 1; No = 0

^2^Appropriate study design: Yes = 1; No = 0

^3^Study conducted appropriately for design: Yes = 1; No = 0

^4^Representativeness of the population: Representative=1; Not representative = 0

^5^Selection of control group: same population = 1; different source = 0

^6^Ascertainment of exposure: collected prospectively = 1; retrospectively = 0

^7^Test for childhood maltreatment: official record =1; self-reported or interview = 0

^8^Assessment of depression: clinical interview = 1; self-report scale = 0

^9^Appropriate statistical analysis: Yes = 1; No = 0

^10^Appropriate methods to control confounding: Multivariable adjustment = 1; Univariate analysis = 0

^11^Analysis results were appropriately interpreted: Yes = 1; No = 0

## Supplementary Figure 1

*Permutation Analysis of the Influence of Individual Studies on the Overall Meta-Analysis Results*

## Supplementary Table 2

### Subgroup Analysis of the of the Association Between Childhood Maltreatment and Adult Depression - Childhood Maltreatment Screening Method

| **CM Test Name** | **Studies** | **Effect estimates** | **Odds Ratio** | **Lower CI** | **Upper CI** | **Weight (%)** | ***I^2^* (%)** | **Q** | ***df*** | ***p*** |
| --- | --- | --- | --- | --- | --- | --- | --- | --- | --- | --- |
| Adverse Childhood Experiences Questionnaire | 21 | 51 | 2.36 | 2.08 | 2.66 | 60.95 | 93.6 | 777.85 | 50 | 0.000 |
| Conflict Tactic Scales | 3 | 3 | 2.15 | 1.58 | 2.92 | 3.36 | 43.7 | 3.55 | 2 | 0.169 |
| Childhood Trauma Questionnaire | 4 | 7 | 3.34 | 1.91 | 5.82 | 6.26 | 91.0 | 66.92 | 6 | 0.000 |
| Interview | 10 | 14 | 2.13 | 1.64 | 2.77 | 15.69 | 90.5 | 136.79 | 13 | 0.000 |
| Newly Created Self-report | 1 | 5 | 2.26 | 1.68 | 3.04 | 10.61 | 90.3 | 82.88 | 8 | 0.000 |

## Supplementary Table 3

### Subgroup Analysis of the of the Association Between Childhood Maltreatment and Adult Depression - Depression Screening Method

| **Depression Test Name** | **Studies** | **Effect estimates** | **Odds Ratio** | **Lower CI** | **Upper CI** | **Weight (%)** | ***I^2^* (%)** | **Q** | ***df*** | ***p*** |
| --- | --- | --- | --- | --- | --- | --- | --- | --- | --- | --- |
| Beck Depression Inventory | 4 | 7 | 2.42 | 1.85 | 3.16 | 8.31 | 82.6 | 34.47 | 6 | 0.000 |
| Centre for Epidemiological Studies Depression | 9 | 17 | 2.11 | 1.72 | 2.59 | 22.36 | 95.4 | 348.94 | 16 | 0.000 |
| Diagnostic Interview | 20 | 34 | 2.27 | 1.95 | 2.66 | 39.81 | 90.6 | 350.79 | 33 | 0.000 |
| Geriatric Depression Scale | 1 | 3 | 5.01 | 2.24 | 11.22 | 1.16 | 0.0 | 0.22 | 2 | 0.897 |
| Patient Health Questionnaire | 7 | 15 | 3.51 | 2.43 | 5.06 | 17.10 | 96.9 | 446.52 | 14 | 0.000 |
| Single Self-report Question | 1 | 9 | 2.65 | 1.83 | 3.82 | 8.27 | 85.0 | 53.41 | 8 | 0.000 |

## Supplementary Figure 2

###
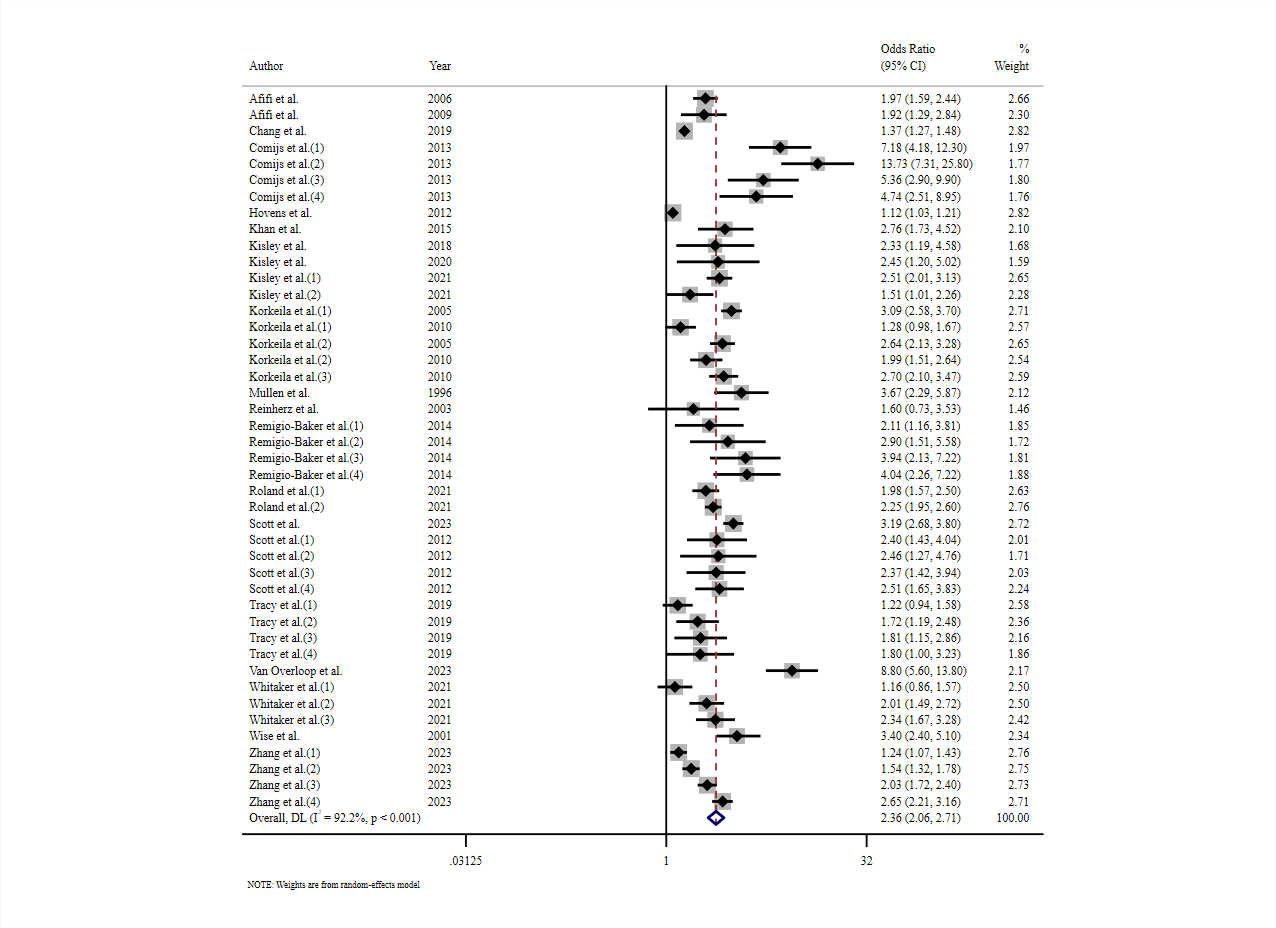
Sensitivity Analysis of High-Quality Studies Examining the Association Between Childhood Maltreatment and Adult Depression
